# Sex-specific and socioeconomic disparities in the global burden of inflammatory bowel disease in 204 countries, 1990–2021: projections to 2050

**DOI:** 10.3389/fimmu.2025.1673212

**Published:** 2026-01-09

**Authors:** Qingtong Li, Yanqi Kou, Mingcheng Zhang, Chunyi Wu, Rongfo Mi, Wantong Jiang, Yufei Cheng, Lei Ge, Ke Yang, Zhe Huang, Yuan Tian, Botao Luo, Yanping Ha, Wanyi Yin, Wei Guo, Wenwei Zhu, Jun Liu, Shicai Ye, Yijie Weng, Jiayuan Wu, Yuping Yang

**Affiliations:** 1 Department of Gastroenterology, Affiliated Hospital of Guangdong Medical University, Guangdong Medical University, Zhanjiang, Guangdong, China; 2 Department of Internal Medicine, Dongguan Maternal and Child Health Care Hospital, Zhanjiang, Dongguan, Guangdong, China; 3 Department of Gastrointestinal Surgery, Affiliated Hospital of Guangdong Medical University, Guangdong Medical University, Zhanjiang Guangdong, China; 4 Department of Colorectal Surgery, Affiliated Hospital of Guangdong Medical University, Zhanjiang, China; 5 Department of Pathology, Guangdong Medical University, Zhanjiang, Guangdong, China; 6 Clinical Research Service Center, Affiliated Hospital of Guangdong Medical University, Zhanjiang, Guangdong, China

**Keywords:** Bayesian age-period-cohort, global burden of disease, inflammatory bowel disease, joinpoint, sex differences, sociodemographic index

## Abstract

**Background:**

Inflammatory bowel disease (IBD) represents a growing global health challenge, necessitating a systematic evaluation of its epidemiological trends and sociodemographic disparities.

**Methods:**

Using Global Burden of Disease 2021 data, we quantified IBD burden (incidence, mortality, prevalence, disability-adjusted life years [DALYs], years lived with disability [YLDs], and years of life lost [YLLs]) across 204 countries and territories from 1990 to 2021, and projected trends to 2050, evaluating associations with Sociodemographic Index (SDI) and sex disparities.

**Results:**

Between 1990 and 2021, global crude counts of incident cases, deaths, prevalence, DALYs, YLDs, YLLs increased by 88.3%, 98.1%, 76.5%, 59.2%, 75.1%, and 50.8%, respectively. However, age-standardized rates (ASRs) of mortality, prevalence, DALYs, YLDs, and YLLs declined by 13.0%, 6.5%, 16.1%, 6.6%, and 21.0%, respectively, contrasting with a modest rise in age-standardized incidence rates (ASIR, 5.5%). The average annual percentage changes (AAPCs) of Incidence, Mortality, Prevalence, DALYs, YLDs, and YLLs were 0.1582, -0.4885, -0.2223, -0.5704, -0.2237, -0.7686, respectively. Projections to 2050 indicate continued reductions in ASRs across all metrics. High and middle-high SDI regions retained the highest absolute burdens, yet low to middle-SDI regions exhibited the steepest escalation in crude incidence and prevalence, correlating strongly with rising SDI (Spearman’s ρ=0.599 for incidence). Females showed a bimodal pattern, with higher disability and prevalence in both mid-life and very old age groups.

**Conclusions:**

High-SDI regions remain IBD epicenters, but accelerating burdens in low- and middle-SDI areas demand context-specific interventions. Persistent sex/age disparities necessitate tailored healthcare strategies to address the evolving global burden.

## 1 Introduction

Inflammatory bowel disease (IBD), encompassing Crohn’s disease and ulcerative colitis, is a chronic immune-mediated disorder of the gastrointestinal tract. It is characterized by relapsing and remitting inflammation that can lead to anemia, strictures, fistulas, and an increased risk of colorectal cancer. These complications often require surgery and impose a substantial and growing global health burden (1, 2).

Over the past decades, IBD has transitioned from being a predominantly Western disease to a global condition. Newly industrialized regions in Asia, South America, and the Middle East have reported rapid increases in incidence and prevalence, paralleling urbanization and lifestyle changes (3), including Taiwan (APC for Crohn’s disease +4.0% [1.0-7.1] and APC for ulcerative colitis +4.8%), Brazil (APC for Crohn’s disease +11.1% [95% CI 4.8-17.8] and APC for ulcerative colitis +14.9% [10.4-19.6]), and other regions where incidence has risen by 1.8–8.0% during 1990–2016 (3–6).

However, critical research gaps persist. Existing burden studies focus predominantly on Western high-income nations, neglecting accelerated disease growth in low-middle SDI regions where healthcare resources are most constrained. While sex differences in IBD burden are acknowledged (7–9), no study has quantified sex-specific disability peaks across age strata, particularly among the mid-life and the very old. Furthermore, for the updated GBD data for 2021, particularly in the context of changing demographic and sociodemographic index (SDI) scenarios, the current global burden of disease across countries and projections of the future burden of disease remain understudied.

This study advances prior GBD-based investigations of IBD in three key ways. First, we incorporate the updated GBD 2021 estimates to provide a comprehensive assessment of IBD burden across 204 countries, 22 regions, and five SDI strata. Second, we quantify sex–age-specific disability profiles for 2021 and identify a distinct bimodal burden pattern among females in mid-life and older adulthood—an observation not reported in earlier GBD analyses. Third, we generate SDI-stratified projections to 2050 using both Nordpred and Bayesian age–period–cohort models, offering the most detailed long-term forecasts of global IBD trends to date.

Our findings reveal a paradox: while crude IBD cases surged globally, age-standardized rates declined modestly. High-SDI regions persistently shoulder the heaviest burden, yet low- to middle-SDI areas face the fastest acceleration. By integrating sex-age-SDI dimensions, this work provides the foundation for equity-driven public health strategies to mitigate IBD’s evolving global footprint.

## 2 Material and methods

### 2.1 Data sources and processing

Epidemiological data on IBD (including Crohn’s disease and ulcerative colitis),were extracted from the Global Health Data Exchange (GHDx) GBD 2021 Results Tool (https://vizhub.healthdata.org/gbd-results/) (10). GBD 2021 is a comprehensive assessment of global health loss that provides up-to-date estimates of the distribution and burden of diseases and injuries by year, age, sex, location, and sociodemographic group (11). These estimates are derived by synthesizing data from vital registration systems, hospital records, population-based surveys, and cohort studies (12). In the GBD framework, the world is divided into 22 epidemiological regions, which are further disaggregated into 204 countries and territories. This classification facilitates the identification of areas with the highest IBD burden and provides a structured basis for targeting intervention strategies.

The definition of cases of inflammatory bowel disease (IBD) was based on the International Classification of Diseases, Tenth Revision (ICD-10) codes K50–K51. The validation of these cases was conducted through a systematic review and meta-regression analysis. Incidence, prevalence, and mortality rates were age-standardized using the GBD reference population to enable cross-regional comparisons. Crude rates were adjusted for population aging using direct standardization.

### 2.2 Age−standardized rates

We calculated age - standardized rates (ASRs) for incidence, prevalence, mortality, YLDs, YLLs, and DALYs using the GBD world standard population. For each location, year, and sex, the ASR was computed as a weighted sum of age-standardized rates:


$$\text{ASR}=\sum_{\text{i}=1}^{\text{n}}{\text{w}}_{\text{i}}\times {\text{r}}_{\text{i}}$$

where ${\text{r}}_{\text{i}}$ is the age-standardized rate in the i-th age group, ${\text{w}}_{\text{i}}$ is the number of individuals in the GBD standard population in the same age group, and i indexes all age groups. This approach removes differences in age structure, enabling valid comparisons over time and between populations.

Crude rates were additionally reported to reflect the absolute burden in the context of population growth and aging.

### 2.3 Joinpoint regression and trend measures

To quantify temporal trends in ASRs from 1990 to 2021, we used Joinpoint regression (Joinpoint Regression Program version 4.9.1.0, National Cancer Institute). For each outcome (ASIR, ASPR, ASMR, age−standardized DALYs, YLDs, YLLs), we modelled the natural logarithm of the ASR as a segmented log-linear function of calendar year:


$$\ln(R_{t})=\beta_{0}+\beta_{1}\times t+\beta 2{\left(t-\tau_{1}\right)}^{+}\;+\ldots \;+\beta_{k+1}{\left(t-\tau_{k}\right)}^{+}$$

Where  t denotes calendar year, $\tau_{k}$ are joinpoints (years where the slope changes), and ${\left(\text{t}-{\text{τ}}_{\text{k}}\right)}^{+}$ denotes the positive part of $\text{t}-{\text{τ}}_{\text{k}}$.We used weighted least-squares estimation with weights equal to the inverse of the variance of each ASR. The maximum number of joinpoints was set to five (i.e. up to six segments), with a minimum segment length of four years.

The number and location of joinpoints were selected using the permutation test procedure implemented in the software, with an overall significance level of 0.05. For each segment, the annual percentage change (APC) was derived from the segment slope ${\text{β}}_{\text{k}}$ as:


$$\;\;APC=(e^{\beta_{k}}-1)\times 100\%$$

and 95% confidence intervals (CIs) for APCs were computed from the standard errors of ${\text{β}}_{\text{k}}$.The average annual percentage change (AAPC) over the entire study period was calculated as a weighted average of segment−specific APCs, with weights proportional to segment length (number of years). CIs for AAPCs were obtained using the delta method and the t distribution, following standard Joinpoint procedures. AAPCs or APCs with 95% CIs not crossing zero were interpreted as statistically significant trends.

The average annual percent change (AAPC) over k segments was obtained as:


$$AAPC=(\prod_{i=1}^{k}{\left(\frac{1+APC_{i}}{100}{)}^{\omega_{i}}\right)}^{\frac{1}{\sum \omega_{i}}}-1$$

### 2.4 Bayesian age–period–cohort projections

To project IBD burden to 2050, we used age–period–cohort (APC) Poisson models at global and SDI-stratified levels. Incidence and prevalence were projected with the nordpred package (R), assuming Poisson counts with a power-5 link and age, period and cohort effects, with drift attenuation in future periods. DALYs, YLDs and YLLs were projected with Bayesian APC models (BAPC package with INLA), using Poisson likelihoods with a log link and smooth second−order random−walk priors for age, period and cohort. All projected rates were age−standardized using the same GBD standard population, and we report medians with 95% prediction/credible intervals. SDI was not included as a covariate in APC models; instead, SDI was used only to define strata and for correlation analyses. Associations between SDI and IBD ASRs were explored using Spearman’s rho and Kendall’s tau. Because BAPC percentiles were generated in 0.002 increments, the 95% credible interval was approximated using the nearest available quantiles (0.024Q and 0.976Q).

### 2.5 SDI classification and correlation analyses

Countries were grouped into five SDI quintiles (low, low-middle, middle, high-middle, high) using GBD 2021 definitions. To explore socioeconomic gradients, we assessed correlations between national SDI values and IBD ASRs (incidence, prevalence, mortality, DALYs, YLDs, YLLs) using Spearman’s rank correlation coefficient and Kendall’s tau.

## 3 Results

### 3.1 Global burden of IBD across all sexes and trends in 2021

Between 1990 and 2021, the global crude incidence of inflammatory bowel disease (IBD) surged from 199,236 cases (95% uncertainty interval [UI]: 174,584–232,676) to 375,140 cases (95% UI: 327,686–436,925; **Supplementary Table S3**), reflecting an 88.3% increase (average annual percentage change [AAPC]: 0.1582; 95% confidence interval [CI]: 0.0728–0.2437; **Table 1**). In contrast, the age-standardized incidence rate (ASIR) exhibited a modest rise from 4.2 per 100,000 (95% UI: 3.7–4.9) to 4.4 per 100,000 (95% UI: 3.9–5.2), representing a percentage change in ASRs of 5.5% (95% CI: 4.0–6.8; **Supplementary Table S3**).

**Table 1:** The AAPCs of incidence, prevalence, mortality, DALYs, YLDs, and YLLs for global IBD by sex from 1990 to 2021.

Metric	Both	Female	Male
	1990 to 2021 AAPC(95%CI)	1990 to 2021 AAPC(95%CI)	1990 to 2021 AAPC(95%CI)
Incidence	0.1582(0.0728,0.2437)	0.1699(0.0529,0.2871)	0.1477(0.0684,0.2271)
Prevalence	-0.2223(-0.2708, -0.1738)	-0.2462(-0.3079,-0.1845)	-0.1905(-0.2467,-0.1343)
Mortality	-0.4885(-0.6848,-0.2917)	-0.526(-0.7398,-0.3118)	-0.3866(-0.5874,-0.1854)
DALYs	-0.5704(-0.6763,-0.4645)	-0.5804(-0.7073,-0.4533)	-0.564(-0.687,-0.4409)
YLDs	-0.2237(-0.2701,-0.1773)	-0.2445(-0.3046,-0.1843)	-0.1989(-0.2535,-0.1442)
YLLs	-0.7686(-0.9487,-0.5883)	-0.8249(-0.9991,-0.6504)	-0.7295(-0.8946,-0.5642)

IBD, inflammatory bowel disease; AAPC: Average Annual Percent Change; CI: Confidence Interval.

Similarly, the global crude prevalence of IBD increased by 76.5%, from 2,170,243 cases (95% UI: 1,892,402–2,522,561; **Supplementary Table S4**) in 1990 to 3,830,119 cases (95% UI: 3,312,834–4,511,555) in 2021. However, the age-standardized prevalence rate (ASPR) declined by 6.5%, decreasing from 48.0 to 44.9 per 100,000 (95% UI: 41.9–55.8 vs. 38.8–52.9; **Supplementary Table S4**), with an AAPC of –0.2223 (95% CI: –0.2708 to –0.1738; **Table 1**).

Mortality trends revealed a pronounced 98.1% increase in crude IBD-related deaths, rising from 21,418 (95% UI: 18,423–23,613) in 1990 to 42,423 (95% UI: 37,537–46,502) in 2021. Notably, the age-standardized mortality rate (ASMR) declined from 0.6 (95% UI: 0.5–0.7) to 0.5 (95% UI: 0.5–0.6) per 100,000 during this period (**Table 2**) (AAPC: –0.4885; 95% CI: –0.6848 to –0.2917; **Table 1**).

**Table 2:** The deaths of IBD, age to standardized mortality of IBD and their percentage change from 1990 to 2021 at the global and regional levels.

Location	1990		2021		1990 to 2021
	no(95% UI)	ASMRs per 100000(95% UI)	2021 No(95% UI)	2021 ASMRs per 100000(95% UI)	Percentage change in the ASMRs per 100000
Global	21418 (18423,23613)	0.6 (0.5,0.7)	42423 (37537,46502)	0.5 (0.5,0.6)	-13 (-21.6,-1.9)
Andean Latin America	60 (44,81)	0.2 (0.2,0.3)	88 (67,112)	0.1 (0.1,0.2)	-34.1 (-50.1,-2.5)
Australasia	73 (67,79)	0.3 (0.3,0.4)	449 (370,504)	0.7 (0.6,0.8)	122.2 (96.3,146.9)
Caribbean	162 (142,184)	0.6 (0.5,0.7)	187 (151,234)	0.4 (0.3,0.4)	-43 (-52.2,-33)
Central Asia	199 (182,218)	0.4 (0.3,0.4)	259 (222,301)	0.3 (0.3,0.4)	-16.8 (-29.6,-1.3)
Central Europe	593 (560,650)	0.4 (0.4,0.5)	864 (788,948)	0.4 (0.4,0.4)	-7.8 (-15.8,1.9)
Central Latin America	312 (301,321)	0.4 (0.3,0.4)	887 (797,976)	0.4 (0.3,0.4)	-0.6 (-9.8,8.9)
Central Sub-Saharan Africa	88 (60,122)	0.4 (0.3,0.6)	222 (146,321)	0.4 (0.3,0.5)	-3.7 (-31.9,26.5)
East Asia	4592 (3149,5758)	0.7 (0.5,0.9)	5967 (4700,7910)	0.3 (0.3,0.4)	-55.7 (-68.3,-30.1)
Eastern Europe	1797 (1655,1976)	0.7 (0.6,0.7)	1827 (1674,1972)	0.5 (0.5,0.6)	-19.5 (-28.9,-9.9)
Eastern Sub to Saharan Africa	322 (228,416)	0.4 (0.3,0.6)	738 (487,996)	0.4 (0.3,0.6)	-3.8 (-20.9,16.9)
High-income Asia Pacific	593 (465,686)	0.3 (0.3,0.4)	670 (520,893)	0.1 (0.1,0.2)	-64.3 (-71.5,-48.3)
High to income North America	2416 (2175,2541)	0.7 (0.6,0.7)	6601 (5760,7049)	1 (0.9,1)	44.8 (39.4,48.9)
North Africa and Middle East	495 (378,714)	0.3 (0.2,0.4)	1055 (857,1389)	0.3 (0.2,0.3)	-11.9 (-30.5,11.5)
Oceania	13 (7,19)	0.4 (0.2,0.6)	21 (14,29)	0.3 (0.2,0.4)	-38.5 (-57.4,6.3)
South Asia	2745 (1905,3528)	0.5 (0.3,0.6)	4718 (3511,6288)	0.3 (0.3,0.5)	-29.8 (-53.6,-1.5)
Southeast Asia	770 (423,1080)	0.3 (0.2,0.5)	1236 (888,1470)	0.2 (0.2,0.3)	-33.5 (-53.4,-1.3)
Southern Latin America	169 (158,179)	0.4 (0.4,0.4)	194 (176,208)	0.2 (0.2,0.2)	-41.6 (-46.2,-36.6)
Southern Sub to Saharan Africa	109 (77,145)	0.4 (0.2,0.5)	210 (163,254)	0.4 (0.3,0.5)	-0.1 (-25.1,34.7)
Tropical Latin America	486 (468,507)	0.5 (0.5,0.5)	1298 (1199,1375)	0.5 (0.5,0.5)	0 (-5.5,5.4)
Western Europe	4577 (4215,4800)	0.8 (0.7,0.8)	12791 (10650,13952)	1.1 (1,1.2)	37.9 (28.9,46.1)
Western Sub-Saharan Africa	845 (582,1121)	0.8 (0.5,1.1)	2140 (1338,2907)	0.8 (0.5,1)	-0.2 (-19.8,24.3)

IBD, inflammatory bowel disease; ASMR, age standardized rate of mortality.

The disability-adjusted life years (DALYs) attributable to IBD demonstrated divergent temporal patterns (AAPC: –0.5704; 95% CI: –0.6763 to –0.4645; **Table 1**). While global crude DALYs rose from 948,861 (95% UI: 808,101–1,096,717) in 1990 to 1,510,784 (95% UI: 1,308,508–1,750,363) in 2021, age-standardized DALY rates per 100,000 population decreased by 16.1% (95% UI: –22.0 to –9.8; **Supplementary Table S5**). A parallel trend was observed for years lived with disability (YLDs): absolute YLDs increased by 75.1% (95% UI: 43.5–112.7), from 330,876 (222,761–456,115) to 579,203 (391,901–799,564), yet the age-standardized YLD rate declined by 6.6% (95% UI: –8.6 to –4.6; **Supplementary Table S1**).

### 3.2 Sex-specific burden of IBD in 2021

#### 3.2.1 Prevalence and incidence

**Figure 1:**
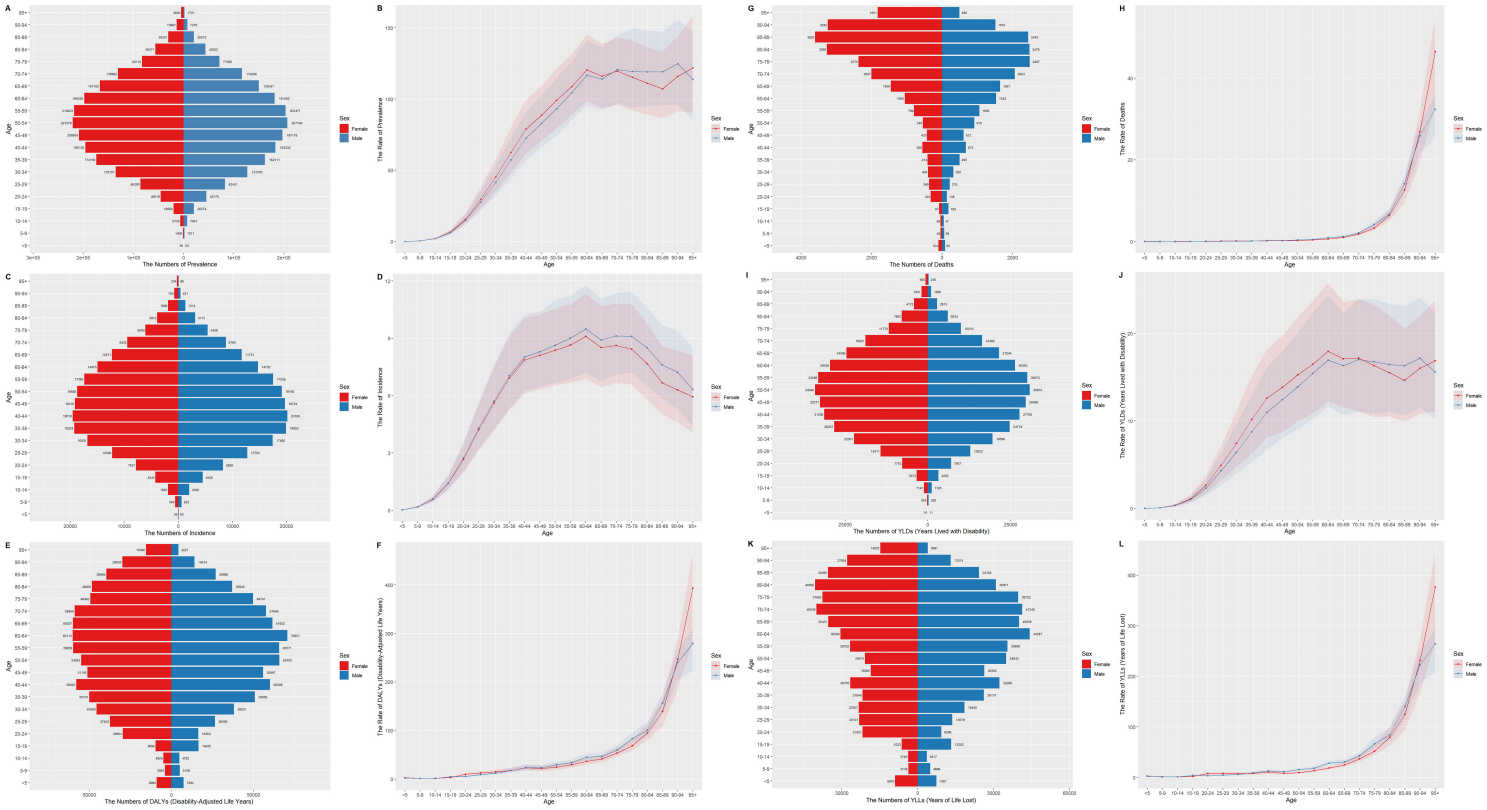
Disease burden of IBD among different sex groups and age groups in 2021 globally. **(A)** The number of prevalence; **(B)** the rate of prevalence; **(C)** the number of incidence; **(D)** the rate of incidence; **(E)** the number of DALYs; **(F)** the rate of DALYs; **(G)** the number of deaths; **(H)** the rate of deaths; **(I)** the number of YLDs; **(J)** the rate of YLDs; **(K)** the number of YLLs; **(L)** the rate of YLLs. IBD, inflammatory bowel disease; DALYs, Disability-Adjusted Life Years; YLDs, Years Lived with Disability; YLLs, Years of Life Lost; ASR, age standardized rate.

In 2021, females exhibited a higher overall burden of inflammatory bowel disease (IBD) compared to males, with distinct age-related patterns across prevalence, mortality, and disability metrics (**Figure 1**). The number of prevalence (**Figures 1A–B**) showed a single, broad peak in mid-life, reaching its maximum at 50–54 years in both sexes, with 221,278 cases among females versus 207,104 cases among males, reflecting a 6.8% female predominance in this critical age stratum. In contrast, the number of incidence peaked earlier, at 40–44 years (**Figures 1C–D**).

#### 3.2.2 Mortality and disability burden by sex

Sex-specific mortality patterns revealed contradictions: although the total number of deaths related to inflammatory bowel disease was higher in females (22,968 vs. 19,455 in males), males had slightly higher mortality rates than females in the middle-aged and older groups (60–89 years). Notably, after 94 years of age, the mortality rate in females exceeded that in males. (**Figure 1G**).

Disability-adjusted life years (DALYs) further underscored these divergences. In the 95+ age group, females bore 3.66 times higher DALYs than males (15,490 vs. 4,227; 95% CI: 3.42–3.91), whereas males predominated in the primary onset age range (45–89 years). Years lived with disability (YLDs) demonstrated pronounced sex differences: females experienced higher YLD rates between ages 20–74, peaking at 17.95 per 100,000 (95% UI: 12.23–25.74) in the 60–64 age group, compared with a male peak of 17.16 (95% UI: 11.52–24.08) at 90–94 years (**Figure 1I**).

#### 3.2.3 Female-specific bimodal patterns

Notably, females displayed bimodal curves in several outcomes. While both sexes demonstrated a decline in the rate of prevalence after middle age, a secondary surge was observed in older populations, particularly among females. In females, the prevalence of IBD peaks at ages 60–64 years and then declines slightly thereafter. But the prevalence rate rose from 107.21 per 100,000 (95% UI: 86.92–133.36) in the 85–89 age group to 115.96 (95% UI: 91.57–144.09) in the 90–94 age group, contrasting with a more gradual increase in males (**Figure 1B**). A similar pattern was observed in the rate of YLDs (**Figure 1L**). In females, YLL rates showed a first peak around 30–44 years, decreased slightly thereafter, followed by a sharp rise around 70–84 years. Men exhibited a more unimodal pattern with fewer late-life fluctuations.

### 3.3 Joinpoint regression analysis of global IBD trends by sex (1990–2021)

#### 3.3.1 Overall trends in disease burden

**Figure 2:**
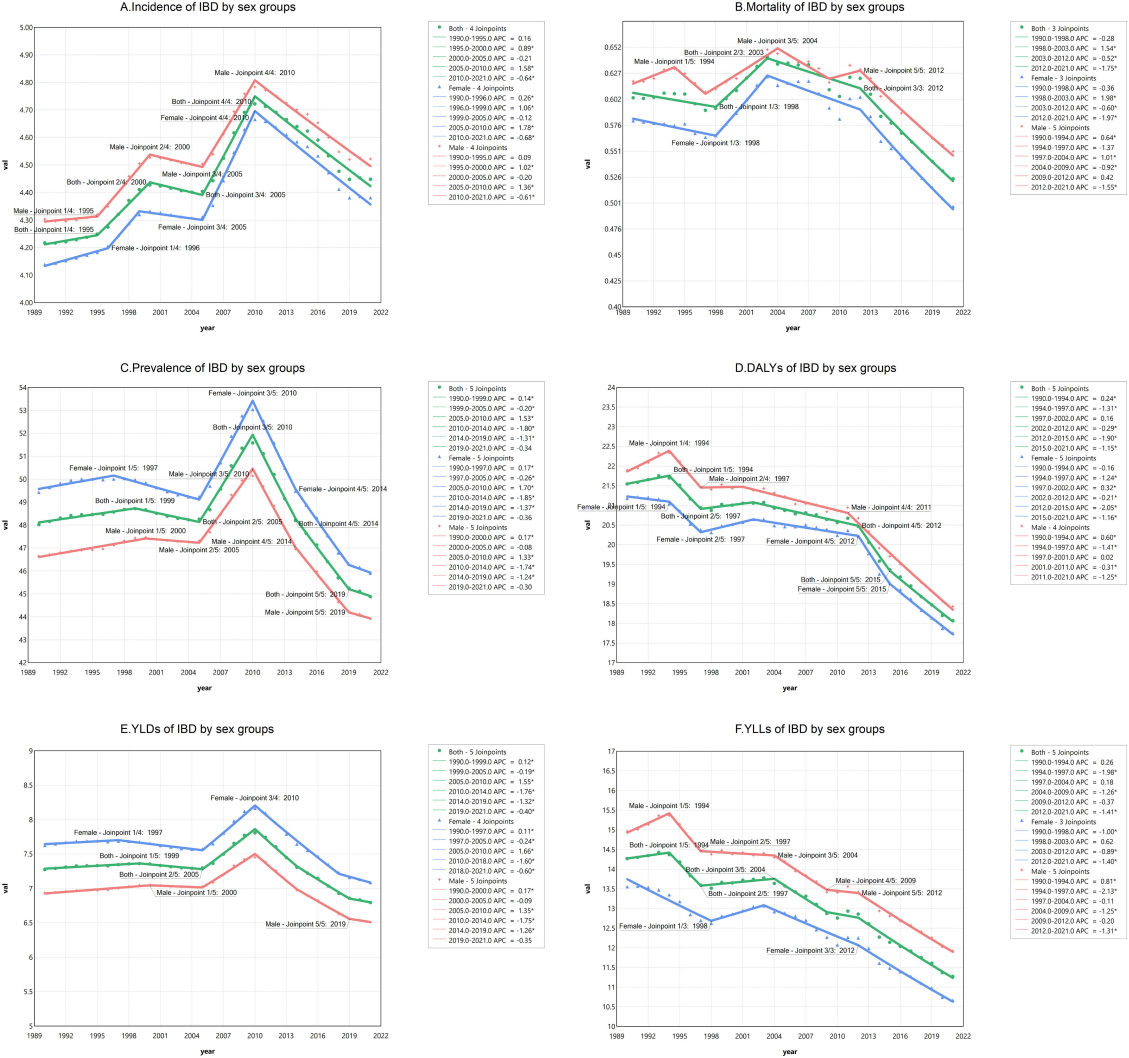
Joinpoint regression analysis of the sex-specific age-standardized rate of incidence, prevalence, mortality, DALYs, YLDs and YLLs for IBD in Global from 1990 to 2021. **(A)** ASIR; **(B)** ASMR; **(C)** ASPR; **(D)** DALYs; **(E)** YLDs; **(F)** YLLs. IBD, inflammatory bowel disease; DALYs, Disability-Adjusted Life Years; YLDs, Years Lived with Disability; YLLs, Years of Life Lost; ASR, age standardized rate.

According to Joinpoint regression analyses, there was a general downward trend in IBD burden from 1990 to 2021 in terms of prevalence (AAPC –0.2223 [95% CI –0.2708 to –0.1738]), mortality (–0.4885 [95% CI –0.6848 to –0.2917]), DALYs (–0.5704 [95% CI –0.6763 to –0.4645]), YLDs (–0.2237 [95% CI –0.2701 to –0.1773]), and YLLs (–0.7686 [95% CI –0.9487 to –0.5883]) (**Figure 2**, **Table 1**).

#### 3.3.2 Incidence trends and sex-specific disparities

Global IBD incidence exhibited dynamic shifts:1990–1995: gradual increase (APC=0.16%; 95% CI: −0.05 to 0.37).1995–2000: acceleration to 0.89% (0.59–1.19), signaling epidemiologic transition.2000–2005: decline (APC=−0.21%; −0.50 to 0.08).2005–2010: rebound to peak growth (APC=1.58%; 1.29–1.88).2010–2021: steep decline (APC=−0.64%; −0.71 to −0.58), with a 76.3% reduction in growth rate.

#### 3.3.3 Sex-stratified analyses highlighted disparities

Males: slower decline post-2010 (APC=−0.61%; p < 0.05) vs. females (APC=–0.68%; –0.75 to –0.61). 1990–2021 cumulative trends: females showed higher overall incidence growth (AAPC=0.17%; 0.05–0.29) compared to males (AAPC=0.15%; 0.07–0.23).

#### 3.3.4 Prevalence and mortality dynamics

Prevalence: A sharp decline occurred from 2010–2014 (APC=−1.80%; −2.00 to −1.61), moderating post-2019 (APC=−0.34%; −0.78 to 0.11). Mortality: after gradual reductions (2003–2012: APC=−0.52%; −0.81 to −0.23), rates plummeted from 2012–2021 (APC=−1.75%; −1.98 to −1.53). Males experienced pronounced fluctuations during 2004–2012.

#### 3.3.5 Disability burden: YLDs and YLLs

YLDs: A transient surge (2005–2010: APC=1.55%; 1.42–1.68) preceded sustained declines post-2010, reflecting improved disease management. YLLs: marked reductions (APC=−0.77%; −0.95 to −0.59) underscored progress in reducing premature mortality. Sex differences emerged:Males: Higher YLLs (1990–1994: APC=0.81%; 0.03–1.65). Females: smaller changes than for men (1990-1998: APC=−1.00%; –1.34 to –0.65).

### 3.4 Global and SDI-stratified trends in IBD burden (1990–2021) and projections to 2050

#### 3.4.1 Methodology and global projections

Using Bayesian Age-Period-Cohort (BAPC) and Nordpred models, we projected age-standardized rates (ASRs) of IBD burden across sociodemographic index (SDI) quintiles. By 2050, global declines are anticipated for incidence (ASIR), prevalence (ASPR), DALYs, and YLDs.

#### 3.4.2 Incidence and mortality dynamics

**Figure 3:**
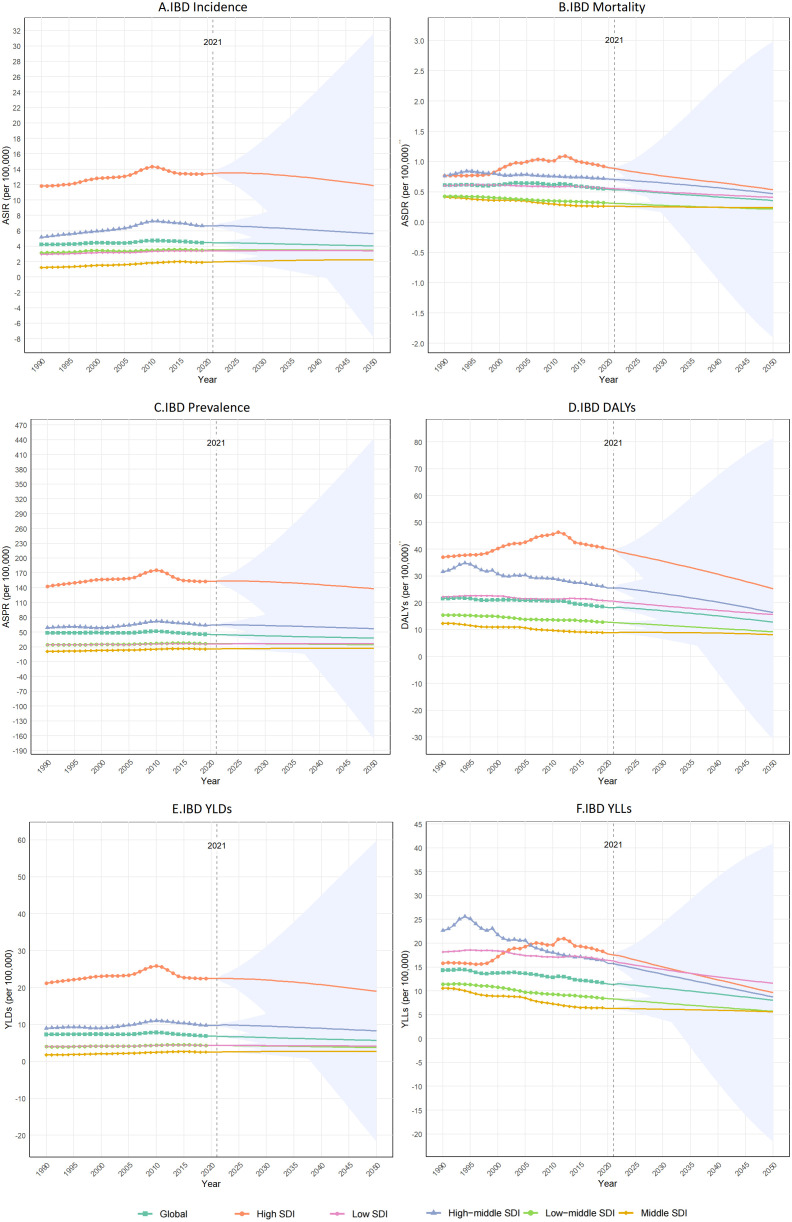
Global and Socio-Demographic Index (SDI)-stratified trends in ASR of incidence, prevalence, mortality, DALYs, YLDs, and YLLs between 1990 and 2021, and peojected changes to 2050. **(A)** ASIR; **(B)** ASMR; **(C)** ASPR; **(D)** DALYs; **(E)** YLDs; **(F)** YLLs. IBD, inflammatory bowel disease; DALYs, Disability-Adjusted Life Years; YLDs, Years Lived with Disability; YLLs, Years of Life Lost; ASR, age standardized rate; SDI, Socio-demographic Index.

Global ASIR is projected to decline from 4.46 per 100,000 in 2021 to 4.04 per 100,000 by 2050. High-SDI regions, however, will retain elevated incidence (11.87 per 100,000 in 2050), reflecting persistent lifestyle and diagnostic patterns. Mortality (ASMR): Despite a transient rise (2000–2015), global ASMR is projected to fall from 0.53 (2021) to 0.36 per 100,000 by 2050. High-SDI regions show the steepest decline (2021: 0.89 per 100,000 → 2050: 0.54 per 100,000) (**Figure 3**; **Supplementary Table S7**).

#### 3.4.3 Prevalence and disability trends

**Figure 4:**
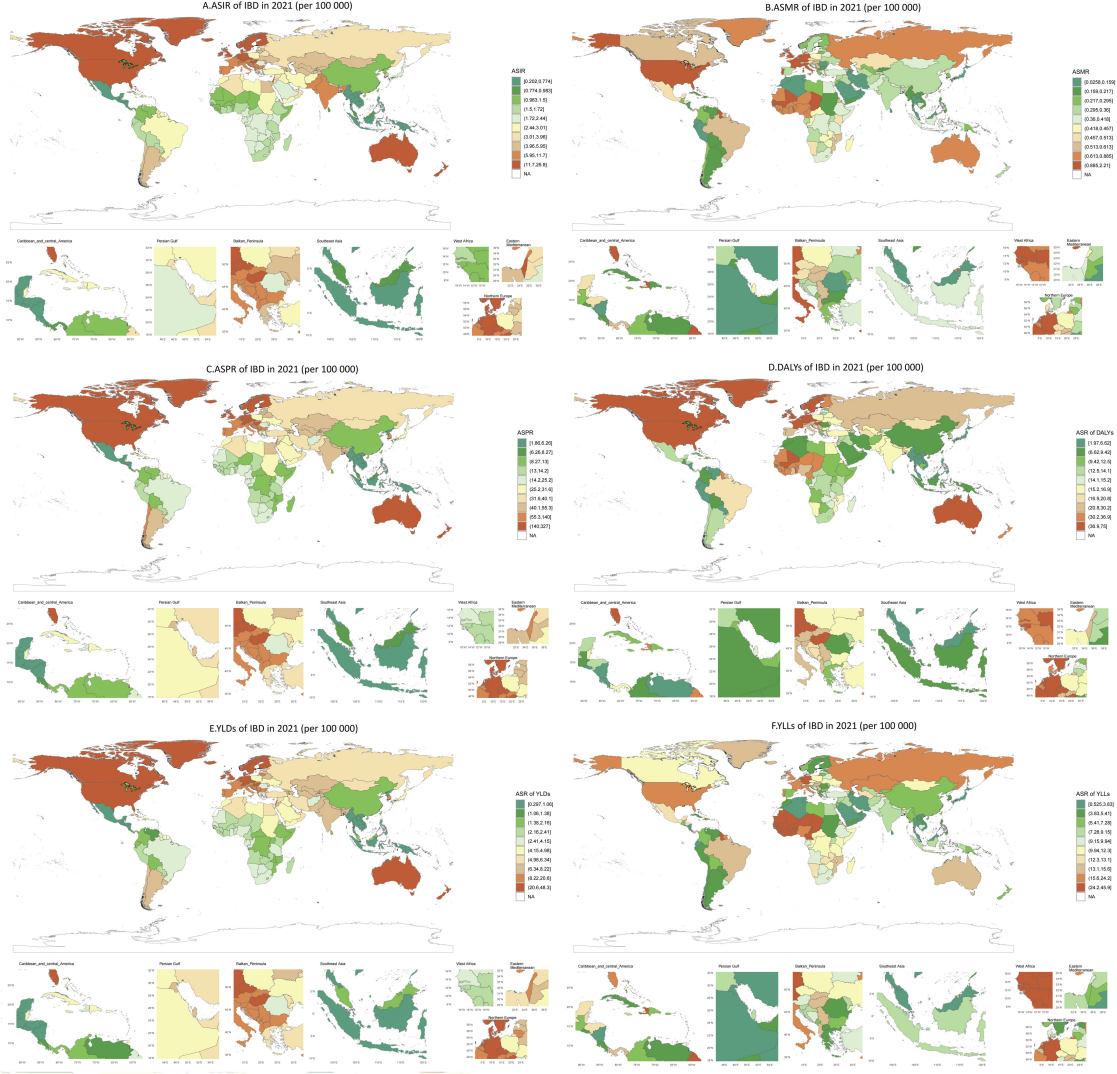
The global disease burden of IBD for both sexes in 204 countries and territories. **(A)** The ASIR of IBD in 2021; **(B)** the ASMR of IBD in 2021; **(C)** the ASPR of IBD in 2021; **(D)** the DALYs of IBD in 2021; **(E)** the YLDs of IBD in 2021; **(F)** the YLLs of IBD in 2021. IBD, inflammatory bowel disease; DALYs, Disability-Adjusted Life Years; YLDs, Years Lived with Disability; YLLs, Years of Life Lost; ASR, age standardized rate.

Global ASPR: Declined by 6.5% from 1990 (48.12/100,000) to 2021 (44.97/100,000), with a projected 15.9% reduction to 37.83/100,000 by 2050. High-SDI regions remain outliers (153.17/100,000 in 2021 → 138.17/100,000 in 2050), while high-middle SDI regions undergo more moderate declines (63.97 → 56.88 per 100,000). DALYs: Global age-standardized DALY rates peaked in 1994 (21.62/100,000) before declining to 18.11/100,000 in 2021, with further reductions projected to 12.82/100,000 by 2050. High-SDI regions exhibited a paradoxical 25.4% rise from 1990–2011 (37.01 → 46.39 per 100,000), followed by a decline to 25.31/100,000 by 2050 (**Figure 4**; **Supplementary Table S7**).

#### 3.4.4 Disability-specific projections

YLDs: Global rates will decrease from 6.81/100,000 (2021) to 5.69/100,000 (2050). High-middle SDI regions show gradual declines (22.42 → 19.01/100,000), while high-SDI areas face volatility (2005–2015), possibly due to survivorship effects. YLLs: The steepest reductions occur in high-SDI regions (17.54 per 100,000 in 2021 → 9.65 per 100,000 in 2050), contrasting with slower progress in middle-SDI areas (6.27 → 5.63 per 100,000). Global YLLs are projected to decline to 8.07 per 100,000 by 2050 (**Figure 4**; **Supplementary Table S7**).

#### 3.4.5 SDI-driven disparities and policy implications

High-SDI Regions: Despite declining rates, absolute burdens remain elevated. Low- and Middle-SDI Regions: Slower mortality declines (e.g., middle-SDI ASMR: −8.09% vs. high-SDI: −39.37%) (**Figure 4**; **Supplementary Table S7**).

### 3.5 SDI-stratified burden of IBD across 22 regions (1990–2021)

#### 3.5.1 Incidence patterns and SDI correlations

A strong positive correlation existed between age-standardized incidence rates (ASIR) and sociodemographic index (SDI) from 1990 to 2021 (Spearman’s ρ=0.599, p < 0.0001). High-income regions dominated the highest ASIRs: Australasia: 19.7 per 100,000 (95% UI: 17.1–23.2), High-income North America: 18.5 per 100,000 (16.3–21.2), Western Europe: 12.7 per 100,000 (11.1–15.0).In contrast, the lowest ASIRs occurred in Central Latin America (0.6 per 100,000 [0.5–0.7]), Southeast Asia (0.7 per 100,000 [0.6–0.8]), and Oceania (0.8 per 100,000 [0.6–0.9]). Notably, East Asia experienced the steepest ASIR increase (86.9% [(81.6–92.0]), while Central Latin America was the only region with declining incidence (−1.8% [−5.4–1.3]). Despite a 57.6% surge (52.8–62.0%), Tropical Latin America’s 2021 ASIR remained low (2.9 per 100,000 [2.5–3.6]) (**Figure 3A**; **Supplementary Table S3**).

#### 3.5.2 Mortality trends and paradoxical SDI links

High-SDI regions exhibited elevated age-standardized mortality rates (ASMR): Western Europe: 1.1 per 100,000 (1.0–1.2). High-income North America: 1.0 per 100,000 (0.9–1.0). Conversely, Andean Latin America (0.1 per 100,000) and High-income Asia Pacific (0.1 per 100,000) reported the lowest ASMRs. Australasia saw the largest ASMR rise (122.2% [96.3–146.9]), yet its 2021 rate (0.7 per 100,000) remained moderate. Strikingly, ASMR and SDI showed no significant correlation (Spearman’s ρ=0.007, p > 0.05)(**Figure 3B**; **Table 2**).

#### 3.5.3 Prevalence dynamics and regional heterogeneity

Age-standardized prevalence rates (ASPR) correlated moderately with SDI (ρ=0.500, p <0.001). In 2021: High-income North America: 198.4 per 100,000, Western Europe: 156.5 per 100,000, Australasia: 203.3 per 100,000. Lowest ASPRs clustered in Southeast Asia (5.7 per 100,000), Central Latin America (5.6 per 100,000), and Oceania (5.6 per 100,000). Sub-Saharan Africa displayed divergent trends: Southern and Western regions saw ASPR rises of 19.7% and 23.9%, respectively, despite minimal SDI progress, while Central Sub-Saharan Africa declined (−5.2% [−6.8–−1.5%]) (**Figure 3C**; **Supplementary Table S4**).

#### 3.5.4 Disability burden: DALYs and YLDs

DALYs exhibited weak but significant SDI correlation (ρ=0.173, p<0.001). High-income North America (49.6 per 100,000 [40.2–60.6]) and Western Europe (42.9 per 100,000 [34.9–52.3]) bore the heaviest burdens, contrasting with East Asia’s steep decline (−57.7% [−68.7–−39.2]). Paradoxically, Western Sub-Saharan Africa’s DALYs (30.2 per 100,000 [19.8–40.7]) exceeded SDI-based expectations, highlighting healthcare disparities (**Figure 3D**; **Supplementary Table S5**).

YLDs showed stronger SDI dependence (ρ=0.467, p < 2.2×10^-16^). High-income North America (28.6 per 100,000) and Australasia (29.7 per 100,000) led globally, yet Tropical Latin America (52.4% [42.7–62.1]) and Western Sub-Saharan Africa (23.7% [20.7–27.1]) experienced disproportionate YLD growth, outpacing SDI advancements (**Figure 3E**; **Supplementary Table S1**).

#### 3.5.5 Premature mortality: YLLs and resource inequity

**Figure 5:**
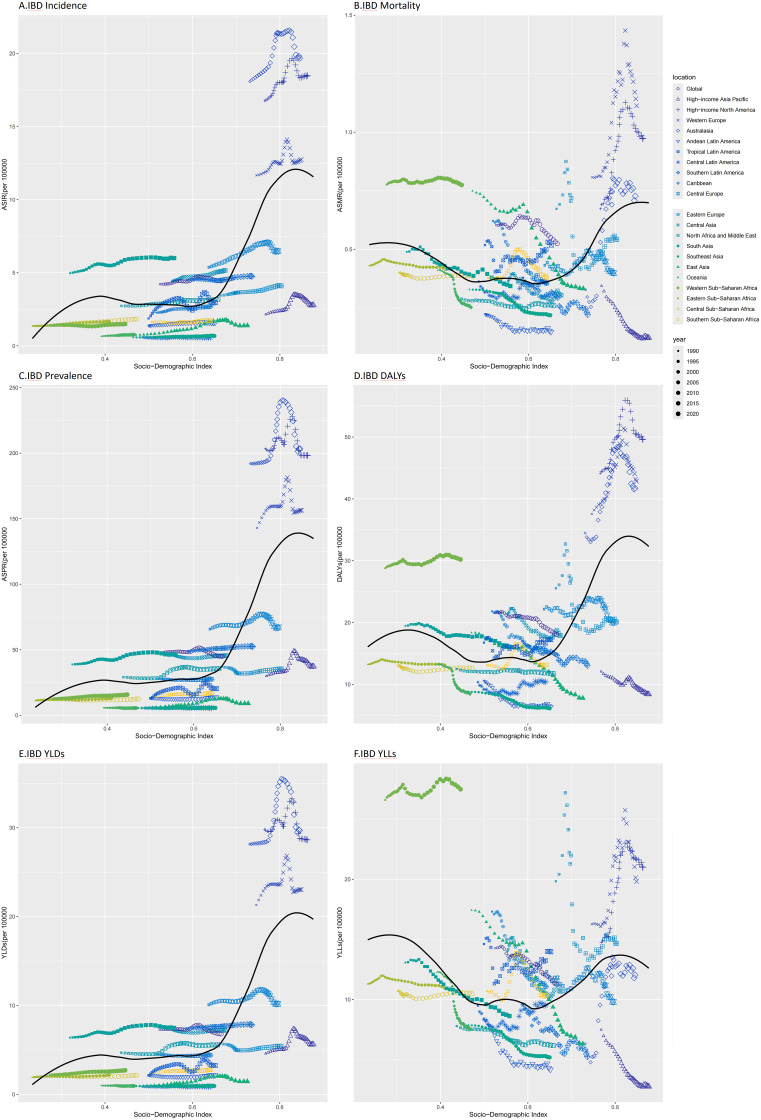
The age-standardized rates based on SDI for 22 GBD regions of IBD Incidence, Mortality, Prevalence, DALYs, YLDs, YLLs globally and from 1990 to 2021. **(A)** ASIR; **(B)** ASMR; **(C)** ASPR; **(D)** DALYs; **(E)** YLDs; **(F)** YLLs. The expected agestandardized rates in 2021 based solely on SDI were represented by the black line. For each region, points from left to right depict estimates from each year from 1990 to 2021. IBD, inflammatory bowel disease; DALYs, Disability-Adjusted Life Years; YLDs, Years Lived with Disability; YLLs, Years of Life Lost; ASR, age standardized rate; SDI, Socio-demographic Index.

Years of life lost (YLLs) were elevated in both low-SDI (e.g., Western Sub-Saharan Africa: 27.5 per 100,000 [17.1–37.5]) and high-SDI regions (e.g., High-income North America: 21.0 per 100,000 [19.5–21.7]). Middle-SDI regions exhibited the lowest YLLs, emphasizing the interplay of healthcare access and survivorship. East Asia achieved the sharpest YLL reduction (−63.8%) (**Figure 5**; **Supplementary Table S2**).

### 3.6 National and global spatial patterns of IBD burden in 204 countries (2021)

#### 3.6.1 Incidence (ASIR) and socioeconomic disparities

A robust positive correlation was observed between age-standardized incidence rates (ASIR) and sociodemographic index (SDI) across 204 countries (Spearman’s ρ=0.599, p <0.0001). High-SDI nations dominated the highest ASIR values: North America/Northern Europe: Canada (26.83 per 100,000), United States (17.59), Sweden (18.86), Denmark (16.30), New Zealand (23.96). Western Europe/Australasia: Greenland (24.57), Netherlands (21.51), Germany (16.64), Australia (18.85).In contrast, low-SDI regions exhibited markedly lower ASIRs: Asia: Philippines (0.53), Vietnam (0.64), North Korea (0.99), China (1.40 [1.21–1.68]).Africa/Mesoamerica: Niger (1.17), South Sudan (1.53), Mexico (0.20), Guatemala (0.66) (**Figure 5A**, **Supplementary Figure S1A**).

#### 3.6.2 Mortality (ASMR) and paradoxical trends

ASMR showed weak inverse correlation with SDI (Spearman’s ρ=−0.090, p=0.212), reflecting mismatched healthcare outcomes. Elevated ASMRs clustered in:High-SDI Europe: Netherlands (2.21 [1.85–2.43]), Germany (1.92 [1.63–2.10]).Low-SDI Caribbean: Somalia (0.38 [0.23–0.56]), Haiti (0.97 [0.55–1.52]). Notably, East Asian nations achieved remarkably low ASMRs: Singapore: 0.026 [0.022–0.028]. Japan: 0.098 [0.086–0.105].China: 0.327 [0.254–0.435], surpassing regional peers (**Figure 5B**; **Supplementary Figure S1B**).

#### 3.6.3 Prevalence (ASPR) and geographic extremes

ASPR correlated strongly with SDI (Spearman’s ρ=0.645, p < 0.0001). The highest ASPRs were concentrated in:High-SDI: Canada (326.64 [278.94–385.56]), Netherlands (278.48 [241.04–327.43]). Europe: Norway (235.77), Sweden (222.48), San Marino (269.34).

Lowest ASPRs occurred in: Latin America: Mexico (1.86 [1.54–2.29]). Southeast Asia: Philippines (4.30 [3.59–5.24]), Cambodia (4.57 [3.83–5.55]) (**Figure 5C**; **Supplementary Figure S1C**).

#### 3.6.4 Disability burden: DALYs and regional inequities

DALYs exhibited a weak SDI correlation (Spearman’s ρ=0.173, p <0.0001), with extremes including: High burden: Germany (70.06 [57.05–84.98]), Netherlands (74.96 [60.11–91.48]). Low burden: Singapore (1.97 [2.59–1.45]), Sri Lanka (2.05 [2.72–1.49]), Malaysia (2.97 [2.38–3.73]), Thailand (3.60 [2.68–4.60]).

Paradoxically, Western Sub-Saharan Africa (e.g., Mali, Niger) reported DALYs exceeding SDI-based expectations (**Figure 5D**; **Supplementary Table S5**).

#### 3.6.5 Disability-specific burden: YLDs and YLLs

YLDs correlated strongly with SDI (ρ=0.635, p <0.0001): High burden: Canada (48.32 [31.81–66.97]), Netherlands (40.88 [26.69–58.01]). Low burden: Mexico (0.30 [0.19–0.43]), Philippines (0.70 [0.46–1.00]). YLLs revealed stark resource-driven disparities:

Sub-Saharan Africa: Mali (42.32 [26.17–63.69]), Gambia (44.00 [28.75–65.55]), Guinea-Bissau (45.93 [29.84–64.78]).

High-SDI outliers: Japan (2.23 [2.07–2.36]), Singapore (0.52 [0.47–0.57]) (**Figure 5**; **Supplementary Table S2**).

### 3.7 Discussion

Compared with previous GBD-based studies of IBD, this study provides new insights into the global epidemiology of IBD by leveraging GBD 2021 data, quantifying sex–age-specific disability burdens, and generating SDI-stratified projections through 2050. These methodological advances allow us to update global patterns, reveal previously unreported sex disparities across age groups, and anticipate future burdens under varying sociodemographic trajectories.

#### 3.7.1 Sex-specific burden and biological mechanisms

We observed distinct sex-specific patterns in the global IBD burden. Females had higher prevalence across much of adulthood and showed a bimodal distribution of DALYs, with peaks in middle age and at very old ages. These findings are consistent with previous reports that IBD incidence and prevalence in women have increased in several regions and that older adults account for a growing share of the IBD burden (female patients: APC: +0.06%, 95% CI: 0.02 - 0.10; older adults: (APC: +0.14%,; 95% CI:0.07 - 0.20%) (8). Similar patterns have been reported across multiple cohort studies. In France, the incidence of Crohn’s disease showed a female predominance(female-to-male ratio: 1.21; P < 0.001) (13) and Denmark (incidence ratio: 1.35; P < 0.0001) (14). Studies from the United States also documented a higher prevalence among women (female-to-male ratio: 1.35) (15). A review of the EPIMAD patient registry further supported this trend, reporting a prevalence ratio of 1.38 (16). A meta-analysis including cohorts from North America, Europe, Australia, and New Zealand found that women aged 25–29 years and those older than 35 had a significantly elevated risk of developing Crohn’s disease (17). Additionally, women with Crohn’s disease often experience more severe clinical symptoms and a greater disease burden than men (average time to disease recurrence after bowel resection: female 4.8 years vs. male 6.5 years, p=0.04; first- or second-degree relative affected by Crohn’s disease: female 15% vs. male 8.3%, p=0.02) (18).

Several explanations may be consistent with these sex and age-specific patterns. Prior immunology and gastroenterology literature suggests that sex hormones, sex-linked immune responses, and differences in gut microbiota could shape susceptibility to chronic inflammatory disorders (19–23). A prospective study reported that, compared with controls, women with IBD experienced gastrointestinal symptoms more frequently during the menstrual cycle, including nausea (30% vs. 7%, p=0.006), bloating (53% vs. 22%, p=0.003), and abdominal pain (68% vs. 38%, p=0.006). Women with IBD also reported more frequent systemic premenstrual symptoms than controls (79% vs. 50%, p=0.003) (24).Clinical evidence suggests that the rise in prostaglandin E_2_ during menstruation can reduce microbiota-dependent intestinal Treg cells, thereby promoting inflammatory activity in IBD (19). Additionally, estrogens have been reported to enhance certain pro−inflammatory pathways, whereas androgens may exert more immunosuppressive effects (22, 25). Autoimmune diseases are also approximately twice as common in women as in men, especially during the reproductive years, and pregnant women with autoimmune conditions have shown increased risks of adverse outcomes (IBD OR 1.57, 95% CI 1.03–2.38) (26, 27). The bimodal DALY curve in females, with peaks at 30–44 and 70–84 years, may reflect cumulative disability from chronic inflammation and age-related frailty (7, 9). Therefore, improving outcomes in patients with IBD will require greater attention to biological sex as a determinant of disease burden, a deeper understanding of its underlying mechanisms, and the development of more individualized treatment strategies. It will also be essential to include more diverse populations in both preclinical and clinical research (26, 28).

Among older adults, we observed markedly increased IBD burden. For example, in women aged 85–89 years, the prevalence reached 107.21 per 100,000 (95% UI 86.92–133.36), increasing to 115.96 per 100,000 (95% UI 91.57–144.09) in those aged 90–94 years. Higher comorbidity burden, frailty, long-term corticosteroid use, and lower uptake of steroid-sparing immunosuppressive therapies may be consistent with the high DALYs observed in very old women and men (29–32). These mechanisms may partly contribute to the earlier and more pronounced burden observed in women and older adults. Future individual-level studies are needed to test these hypotheses.

#### 3.7.2 Divergent trajectories across SDI strata and limitations of SDI

Our results show clear SDI-related gradients in IBD burden. High-SDI regions, including Western Europe, high-income North America and Australasia, still have the highest age-standardized incidence and prevalence. IBD is increasing in Europe (0.2% of the Europeans suffer from IBD in 2020) (33), and an East–West gradient exists in Europe (34). In other article, high SDI countries accounted for approximately half of the global prevalent cases and incident-onset cases of elderly IBD in 2019 (29, 35). Higher values in countries such as Denmark (16.30/100,000), may be related to environmental factors (36, 37). Higher intake of ultra-processed food may be positively associated with risk of IBD in high-income regions, while the rhythmicity is associated with the development of Crohn’s disease (38, 39).A case–control study from the Netherlands that examined sex-stratified dietary behaviors in 207 patients with ulcerative colitis found that, compared with controls, individuals with ulcerative colitis consumed less total protein, animal protein, carbohydrates, and alcohol, but had higher overall fat intake. In addition, genetic factors may also contribute. A meta-analysis provided evidence that the TLR4 Asp299Gly polymorphism is associated with an increased risk of both Crohn’s disease and overall inflammatory bowel disease (40).

We also identified regional and national outliers where IBD burden is higher or lower than expected for the SDI level. Crude incidence and prevalence are rising rapidly from a lower baseline in many parts of Asia, Latin America, and North Africa. Regions such as Central Sub-Saharan Africa show rising incidence with limited improvements in ASMR and DALYs, likely reflecting diagnostic gaps, late presentation, and competing causes of death. These apparent paradoxes may reflect a combination of genetic background, local environmental exposures, health-system structure, and data limitations, including under-reporting (41, 42). Low, Low-middle, and Middle SDIs should take notice of the escalating IBD burden within their regions (43, 44).

Importantly, SDI is an aggregate index of income, education, and fertility, and it does not capture healthcare quality, dietary changes, urbanization, or environmental exposures, all of which are relevant to IBD. Differences in health-system organization, insurance coverage, antibiotic use, and pollution may therefore confound SDI–IBD gradients and help explain heterogeneity between regions with similar SDI (21).

#### 3.7.3 Regional paradoxes and unresolved drivers

We also identified outliers that do not follow the expected SDI gradient. Japan is a high-SDI country but has relatively low ASIR and ASMR compared with other high-income settings (ASIR: 1.94/100,000). This pattern may reflect a combination of genetic background, traditional dietary habits with higher fish and fiber intake, and strong primary care and screening programs that support early detection and good long-term management of gastrointestinal diseases (45, 46) In contrast, small European microstates such as San Marino show very high modelled prevalence and incidence compared with neighboring countries (47). In such small populations, a limited number of cases can lead to large swings in age-standardized rates. Differences in registry completeness, diagnostic thresholds and coding practices can further amplify these apparent deviations.

These examples underline the need to interpret GBD estimates together with information on data quality and coding. In many low- and middle-SDI countries, including parts of Latin America (+57.6%) and Sub-Saharan Africa, GBD relies mainly on sparse or hospital-based data (48). Some apparently paradoxical patterns—such as low recorded prevalence but rapidly rising crude incidence in urbanizing Latin America, or increasing ASPR in Central Sub-Saharan Africa despite low SDI—therefore likely reflect both real epidemiological transitions and modeling artefacts (44, 49). Building population-based IBD registries, harmonizing diagnostic criteria and improving coding completeness will be essential to separate true signals from data limitations.

To address the disease burden in low-SDI regions, targeted healthcare strategies could be implemented. For example, using MM, TPMT, and TPMT+MM strategies may lead to earlier responses and lower costs compared to CC alone (time to initial response: 18.66, 18.96, and 19.10 weeks vs. 22.41 weeks; duration of response: 39.83, 42.91, and 39.80 weeks vs. 45.36 weeks) (50, 51). Additionally, care could be strengthened by managing stable patients in primary care, supporting consultation between gastroenterologists and local clinicians, and using nurses to coordinate medication, testing, and specialized IBD clinics for high-need patients.

#### 3.7.4 The mortality–prevalence paradox

A key finding of our study is the mismatch between SDI and different IBD indicators. Higher SDI is strongly associated with higher incidence (ρ=0.599) and prevalence (ρ=0.645). However, we observed almost little to no correlation between SDI and IBD-specific ASMR (Spearman’s ρ=0.007, p > 0.05).

From an epidemiological perspective, prevalence reflects both incidence and duration of disease. High-SDI regions have long had high or stable incidence, but also benefit from earlier diagnosis, better access to effective therapies and improved management of complications (1, 5, 22). Patients therefore live longer with IBD, which increases prevalence but does not necessarily raise IBD-coded mortality. In addition, deaths among people with IBD in high-SDI settings are often attributed to gastrointestinal cancer, COVID-19, infection or cardiovascular disease rather than to IBD itself (52, 53). This practice lowers ASMR even when the underlying burden remains substantial.

In low-SDI settings, limited availability of endoscopy, histopathology and specialist care leads to underdiagnosis and misclassification. Many deaths that are causally related to IBD may be recorded as infectious colitis, intestinal tuberculosis or malnutrition (48). Sub-Saharan Africa’s rising ASPR amid low SDI may be consistent with deficiencies in diagnostic and clinical capacity, as well as a substantial rate of misdiagnosis in the context of a high burden of infectious diseases (44). In such contexts, both recorded prevalence and ASMR can be low despite a considerable true burden.

Regional examples in our data fit this framework. In East Asia, ASMR fell by more than half between 1990 and 2021 (43). Over the same period, health insurance coverage, diagnostic capacity and access to effective treatments all improved [e.g. the ASR of death and DALYs had significantly decreased (EAPC: −3.05 and −2.93, respectively)] (54–56), These changes likely reduced deaths attributed to IBD, even as incidence rose and more patients survived with chronic disease. China and San Marino show further discordance. China has declining mortality indicators despite increasing prevalence, while San Marino has very high modelled prevalence but only modest mortality (47).

#### 3.7.5 Limitations

This study has several limitations. First, all estimates rely on the GBD 2021 modelling framework, and data quality varies across countries. In settings with sparse primary data, estimates may therefore depend heavily on modelled rather than observed values. Second, Crohn’s disease and ulcerative colitis were combined as “IBD”, so subtype-specific patterns, such as earlier onset in Crohn’s disease or differences in extraintestinal manifestations, could not be assessed. Third, we examined multiple correlations between SDI and several IBD outcomes across regions and countries. Because we tested multiple correlations between SDI and several IBD outcomes without formal adjustment for multiple comparisons, some statistically significant associations may have occurred by chance (type I error), and these findings should therefore be interpreted as exploratory.

Despite these limitations, the study provides an updated and globally comparable overview of IBD burden using the most recent GBD 2021 data and offers sex-, age-, and SDI-specific insights that can support future research and health-system planning.

## 4 Conclusion

While high-SDI regions remain epicenters of IBD burden, accelerating trends in low- and middle-SDI areas underscore an urgent need for context-specific interventions. Persistent sex- and age-related disparities highlight the importance of tailored healthcare strategies across the life course.

## Data Availability

The original contributions presented in the study are included in the article/**Supplementary Material**. Further inquiries can be directed to the corresponding authors.
