# Supplementary material for: Sex-specific and socioeconomic disparities in the global burden of inflammatory bowel disease in 204 countries, 1990–2021: projections to 2050
Source: Front Immunol. 2026 Jan 9;16:1673212. doi: 10.3389/fimmu.2025.1673212 (PMC12827582; doi:10.3389/fimmu.2025.1673212)
Supplement: Supplementary file 1 [file Table1.docx]

**Figure S1**

The age-standardized rates based on SDI for 204 territories of IBD Incidence, Mortality, Prevalence, DALYs, YLDs, YLLs in 2021. (A) ASIR; (B) ASMR; (C) ASPR; (D) DALYs; (E) YLDs; (F) YLLs. The expected agestandardized rates in 2021 based solely on SDI were represented by the black line. For each region, points from left to right depict estimates from each year from 1990 to 2021.

Abbreviations: IBD, inflammatory bowel disease; DALYs, Disability-Adjusted Life Years; YLDs, Years Lived with Disability; YLLs, Years of Life Lost; ASR, age standardized rate; SDI, Socio-demographic Index

**
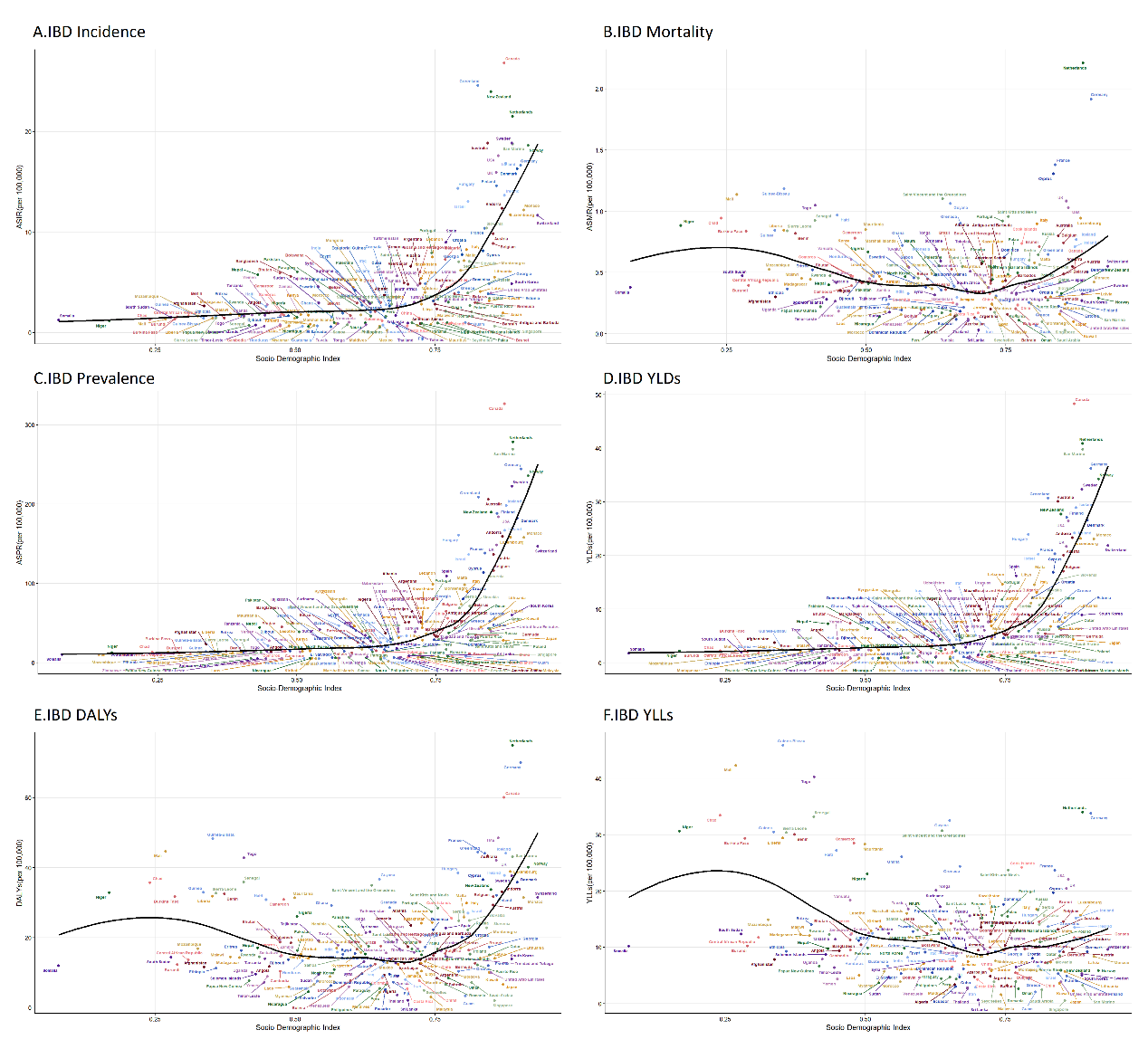
**

**TableS1.The Years Lived with Disability (YLDs) of IBD , age to standardized prevalence rate of IBD and their Percentage change from 1990 to 2021 at the global and regional levels.**

| **Location** | **1990** | |  | **1990 to 2021** | |  | **1990 to 2021** |
| --- | --- | --- | --- | --- | --- | --- | --- |
|  | **no(95% UI)** | **ASRs of YLDs per 100000(95% UI)** |  | **2021 No(95% UI)** | **ASRs of YLDs per 100000(95% UI)** |  | **Percentage change in the YLDs per 100000** |
| Global | 330876 (222761,456115) | 7.3 (4.9,10) |  | 579203 (391901,799564) | 6.8 (4.6,9.3) |  | -6.6 (-8.6,-4.6) |
| Andean Latin America | 602 (390,862) | 2.2 (1.4,3.1) |  | 1414 (910,2052) | 2.2 (1.4,3.1) |  | -0.2 (-9.6,9) |
| Australasia | 6275 (4128,8874) | 28.2 (18.5,39.8) |  | 11689 (7907,16150) | 29.7 (19.8,41.8) |  | 5.5 (-2.7,14.7) |
| Caribbean | 1365 (895,1940) | 4.5 (2.9,6.4) |  | 2272 (1509,3238) | 4.4 (2.9,6.3) |  | -2 (-8.1,4.6) |
| Central Asia | 4041 (2671,5786) | 7.3 (4.9,10.5) |  | 7006 (4710,10043) | 7.2 (4.8,10.3) |  | -1.9 (-9.4,4.5) |
| Central Europe | 14038 (9381,19832) | 10.1 (6.7,14.2) |  | 15382 (10398,21627) | 10.2 (6.7,14.3) |  | 1.2 (-3.2,5.4) |
| Central Latin America | 1053 (682,1497) | 0.9 (0.6,1.2) |  | 2301 (1484,3332) | 0.9 (0.6,1.3) |  | -1.2 (-9.1,8.1) |
| Central Sub-Saharan Africa | 777 (508,1098) | 2.3 (1.5,3.2) |  | 1944 (1242,2761) | 2.1 (1.4,3.1) |  | -7.5 (-14.4,-0.3) |
| East Asia | 10591 (6958,15264) | 0.9 (0.6,1.3) |  | 27593 (18358,39346) | 1.5 (1,2.1) |  | 58.2 (52.4,64.2) |
| Eastern Europe | 15050 (10215,21384) | 5.7 (3.9,8.1) |  | 15381 (10258,21993) | 5.4 (3.6,7.7) |  | -4.9 (-9.8,0) |
| Eastern Sub to Saharan Africa | 2132 (1406,3056) | 1.9 (1.3,2.7) |  | 5877 (3872,8414) | 2.2 (1.4,3.1) |  | 12.7 (9.9,15.5) |
| High-income Asia Pacific | 9150 (6061,12870) | 4.6 (3,6.5) |  | 15436 (10235,21497) | 5.6 (3.7,7.9) |  | 22.5 (14,31.1) |
| High to income North America | 94811 (64109,130891) | 29.8 (20.2,41.2) |  | 142210 (97050,195467) | 28.6 (19.1,39.7) |  | -4 (-7.9,-0.2) |
| North Africa and Middle East | 11423 (7583,15998) | 4.7 (3.1,6.6) |  | 33799 (22449,47650) | 5.6 (3.7,7.8) |  | 18.9 (12.8,25.3) |
| Oceania | 48 (32,71) | 1 (0.7,1.4) |  | 110 (72,160) | 1 (0.6,1.4) |  | -4.4 (-10.6,2.8) |
| South Asia | 49899 (33342,70605) | 6.4 (4.3,9) |  | 130141 (87747,182711) | 7.5 (5.1,10.5) |  | 16.8 (13,21) |
| Southeast Asia | 3530 (2327,5096) | 0.9 (0.6,1.3) |  | 7079 (4691,10146) | 0.9 (0.6,1.4) |  | 0.8 (-2.2,3.8) |
| Southern Latin America | 3506 (2275,5043) | 7.4 (4.8,10.7) |  | 6089 (3948,8810) | 7.8 (5,11.3) |  | 5.5 (-6.3,17.7) |
| Southern Sub to Saharan Africa | 854 (568,1210) | 2.4 (1.6,3.4) |  | 1993 (1329,2851) | 2.8 (1.9,4) |  | 17 (12.8,21.5) |
| Tropical Latin America | 2648 (1752,3711) | 2.1 (1.4,3) |  | 8430 (5625,11895) | 3.3 (2.2,4.6) |  | 52.4 (42.7,62.1) |
| Western Europe | 96449 (64986,132353) | 21.3 (14.2,29.4) |  | 134978 (90808,188510) | 23 (15.1,32.1) |  | 8.2 (2.7,14.1) |
| Western Sub-Saharan Africa | 2634 (1741,3752) | 2.2 (1.5,3.1) |  | 8080 (5370,11539) | 2.7 (1.8,3.8) |  | 23.7 (20.7,27.1) |

Abbreviations: IBD, inflammatory bowel disease; YLDs, Years Lived with Disability; ASR, age standardized rate.

**TableS2.The Years of Life Lost (YLLs) of IBD , age to standardized YLLs rate of IBD and their Percentage change from 1990 to 2021 at the global and regional levels.**

| **Location** | **1990** | |  | **1990 to 2021** | |  | **1990 to 2021** |
| --- | --- | --- | --- | --- | --- | --- | --- |
|  | **no(95% UI)** | **ASRs of YLLs per 100000(95% UI)** |  | **2021 No(95% UI)** | **ASRs of YLLs per 100000(95% UI)** |  | **Percentage change in the YLLs per 100000** |
| Global | 617984 (525972,701005) | 14.3 (12.2,15.9) |  | 931581 (827969,1023099) | 11.3 (10,12.4) |  | -21 (-28.3,-12.1) |
| Andean Latin America | 3108 (2053,4515) | 8.4 (5.9,11.6) |  | 2642 (1992,3398) | 4.2 (3.1,5.3) |  | -50.4 (-66.4,-21.6) |
| Australasia | 1431 (1327,1535) | 6.3 (5.9,6.8) |  | 6493 (5568,7233) | 12 (10.6,13.3) |  | 90.5 (70.7,110.4) |
| Caribbean | 5110 (4295,6061) | 17.3 (14.8,20.2) |  | 5649 (4367,7367) | 11.1 (8.5,14.4) |  | -36.1 (-47.9,-23.3) |
| Central Asia | 9024 (7605,10137) | 14 (12.4,15.6) |  | 10033 (8504,11804) | 10.7 (9.1,12.5) |  | -23.8 (-38,-4.4) |
| Central Europe | 16638 (15796,18184) | 12.1 (11.4,13.2) |  | 18355 (16823,20181) | 9.8 (8.9,10.8) |  | -19 (-26.6,-9.6) |
| Central Latin America | 10719 (10337,11093) | 9.5 (9.2,9.7) |  | 24465 (22082,27080) | 9.5 (8.6,10.5) |  | 0.5 (-8.9,11.1) |
| Central Sub-Saharan Africa | 3492 (2267,4905) | 10.7 (7.3,15) |  | 8732 (5576,12651) | 10.5 (6.9,15.1) |  | -2.3 (-31,35.4) |
| East Asia | 156905 (110033,202335) | 17.4 (12,22.1) |  | 115975 (91588,151613) | 6.3 (5,8.1) |  | -63.8 (-73.6,-46.7) |
| Eastern Europe | 52475 (48265,58924) | 19.8 (18.2,22.3) |  | 45403 (41928,49055) | 14.7 (13.5,15.8) |  | -26 (-36,-16.4) |
| Eastern Sub to Saharan Africa | 12015 (8795,15535) | 11.3 (7.8,14.7) |  | 27659 (17978,36951) | 11 (7.2,14.8) |  | -3 (-26.7,24) |
| High-income Asia Pacific | 13789 (10800,15735) | 7.5 (5.8,8.6) |  | 10642 (8789,13713) | 2.8 (2.4,3.5) |  | -63 (-70.7,-47.2) |
| High to income North America | 48297 (45397,49965) | 14.3 (13.5,14.8) |  | 124061 (113523,129676) | 21 (19.5,21.7) |  | 46.5 (42.6,50.1) |
| North Africa and Middle East | 18452 (12725,29826) | 7.8 (5.9,11.4) |  | 31436 (25206,41861) | 6.2 (5,8.2) |  | -20.6 (-39.9,8.8) |
| Oceania | 503 (258,728) | 12.2 (6.3,17.9) |  | 774 (525,1114) | 7.5 (5.1,10.7) |  | -38.6 (-58.6,9.4) |
| South Asia | 96379 (67555,131439) | 13.1 (9.1,16.8) |  | 139222 (103767,184844) | 8.6 (6.4,11.4) |  | -34 (-51.8,-12.3) |
| Southeast Asia | 24944 (14654,32140) | 7.9 (4.4,10.8) |  | 33587 (24413,40122) | 5.2 (3.7,6.2) |  | -34.1 (-49.3,-8) |
| Southern Latin America | 4480 (4203,4733) | 9.6 (9,10.1) |  | 4332 (4054,4600) | 5.2 (4.9,5.6) |  | -45.4 (-49.7,-40.5) |
| Southern Sub to Saharan Africa | 4120 (3151,5065) | 10.7 (7.7,13.9) |  | 6969 (5530,8476) | 10.3 (8.1,12.5) |  | -3.5 (-24.4,23.9) |
| Tropical Latin America | 16768 (16253,17359) | 14.6 (14.1,15.2) |  | 35483 (33504,37189) | 14 (13.2,14.6) |  | -4.4 (-9.2,1.2) |
| Western Europe | 84175 (79857,87124) | 16.3 (15.5,16.8) |  | 183258 (159645,196194) | 19.8 (18,21) |  | 21.8 (15.3,28.8) |
| Western Sub-Saharan Africa | 35162 (23256,44284) | 26.6 (18.1,34.8) |  | 96411 (57177,133632) | 27.5 (17.1,37.5) |  | 3.4 (-16.8,29.8) |

Abbreviations: IBD, inflammatory bowel disease;YLDs, Years Lived with Disability; YLLs: Years of Life Lost; ASR, age standardized rate.

**TableS3.The incidence of IBD,age to standardized incidence rate of IBD and their Percentage change from 1990 to 2021 at the global and regional levels.**

| **Location** | **1990** | |  | **2021** | |  | **1990-2021** |
| --- | --- | --- | --- | --- | --- | --- | --- |
|  | **no(95% UI)** | **ASIRs per 100000(95% UI)** |  | **2021 No(95% UI)** | **2021 ASIRs per 100000(95% UI)** |  | **Percentage change in the ASIRs per 100000** |
| Global | 199236 (174584,232676) | 4.2 (3.7,4.9) |  | 375140 (327686,436925) | 4.4 (3.9,5.2) |  | 5.5 (4,6.8) |
| Andean Latin America | 399 (342,483) | 1.4 (1.2,1.6) |  | 1050 (896,1298) | 1.6 (1.4,2) |  | 16.9 (12.2,22) |
| Australasia | 4002 (3422,4743) | 18.1 (15.5,21.4) |  | 7307 (6372,8465) | 19.7 (17.1,23.2) |  | 8.7 (4.2,14.1) |
| Caribbean | 727 (617,881) | 2.3 (1.9,2.7) |  | 1379 (1182,1660) | 2.7 (2.3,3.3) |  | 19.7 (16.1,23.1) |
| Central Asia | 2524 (2178,3067) | 4.4 (3.8,5.3) |  | 5018 (4317,6101) | 5.2 (4.5,6.2) |  | 17.1 (14.2,20.3) |
| Central Europe | 7318 (6410,8623) | 5.4 (4.7,6.3) |  | 9297 (8128,10754) | 6.5 (5.6,7.7) |  | 20.6 (18,23.5) |
| Central Latin America | 739 (626,904) | 0.6 (0.5,0.7) |  | 1502 (1263,1849) | 0.6 (0.5,0.7) |  | -1.8 ( -5.4,1.3) |
| Central Sub-Saharan Africa | 505 (433,616) | 1.4 (1.2,1.6) |  | 1751 (1488,2156) | 1.8 (1.6,2.2) |  | 33.6 (27.3,39.7) |
| East Asia | 8633 (7284,10588) | 0.7 (0.6,0.9) |  | 25532 (22107,30530) | 1.4 (1.2,1.7) |  | 86.9 (81.6,92) |
| Eastern Europe | 8896 (7706,10721) | 3.4 (3,4.1) |  | 11229 (9681,13550) | 4.1 (3.6,4.9) |  | 19.4 (16.7,21.8) |
| Eastern Sub to Saharan Africa | 1642 (1407,2000) | 1.4 (1.2,1.6) |  | 4903 (4178,5999) | 1.7 (1.5,2) |  | 23.3 (21.1,25.4) |
| High-income Asia Pacific | 4179 (3633,4948) | 2.2 (1.9,2.6) |  | 6531 (5695,7659) | 2.8 (2.4,3.3) |  | 29.1 (25.2,33.2) |
| High to income North America | 52440 (46626,59946) | 16.8 (14.9,19.2) |  | 84757 (75246,95386) | 18.5 (16.3,21.2) |  | 10.4 (7.5,13.1) |
| North Africa and Middle East | 7180 (6129,8700) | 2.7 (2.3,3.3) |  | 19656 (16586,24187) | 3.2 (2.7,3.9) |  | 16.3 (13.4,18.9) |
| Oceania | 33 (28,41) | 0.6 (0.6,0.8) |  | 90 (76,112) | 0.8 (0.6,0.9) |  | 17 (11.1,23.4) |
| South Asia | 40231 (34456,48840) | 5 (4.3,6) |  | 106215 (91605,128247) | 6 (5.2,7.2) |  | 20.5 (19.1,22.3) |
| Southeast Asia | 2268 (1922,2798) | 0.6 (0.5,0.7) |  | 5035 (4271,6181) | 0.7 (0.6,0.8) |  | 19 (17.2,21.3) |
| Southern Latin America | 2152 (1853,2632) | 4.5 (3.9,5.5) |  | 3615 (3128,4385) | 4.7 (4.1,5.8) |  | 5.5 (0.3,10.9) |
| Southern Sub to Saharan Africa | 630 (539,762) | 1.6 (1.4,2) |  | 1285 (1101,1551) | 1.7 (1.5,2) |  | 4.9 (2.7,7.2) |
| Tropical Latin America | 2362 (2030,2878) | 1.9 (1.6,2.3) |  | 7616 (6550,9287) | 2.9 (2.5,3.6) |  | 57.6 (52.8,62) |
| Western Europe | 50598 (45346,57683) | 11.7 (10.4,13.4) |  | 66438 (58285,76430) | 12.7 (11.1,15) |  | 9.3 (5,13.8) |
| Western Sub-Saharan Africa | 1778 (1527,2150) | 1.4 (1.2,1.6) |  | 4933 (4236,5971) | 1.5 (1.3,1.8) |  | 8.7 (7.4,10.2) |

Abbreviations: IBD, inflammatory bowel disease; ASIR, age standardized rate of incidence.

## TableS4.The prevalence of IBD,age to standardized prevalence rate of IBD and their Percentage change from 1990 to 2021 at the global and regional levels.

| **Location** | **1990** | |  | **2021** | |  | **1990 to 2021** |
| --- | --- | --- | --- | --- | --- | --- | --- |
|  | **no(95% UI)** | **AS**PRs per 1**00000(95% UI)** |  | **2021 No(95% UI)** | **ASPRs per 100000(95% UI)** |  | **Percentage change in the ASPRs per 100000** |
| Global | 2170243 (1892402,2522561) | 48 (41.9,55.8) |  | 3830119 (3312834,4511555) | 44.9 (38.8,52.9) |  | -6.5 (-8.4,-5) |
| Andean Latin America | 3642 (3075,4381) | 13.2 (11.2,15.8) |  | 8917 (7559,10768) | 13.6 (11.6,16.4) |  | 3.6 (-1.1,8.7) |
| Australasia | 42856 (36689,51494) | 192 (164.1,230.8) |  | 80697 (69821,95000) | 203.3 (174.7,240.9) |  | 5.9 (0.6,11.3) |
| Caribbean | 8408 (7020,10028) | 27.9 (23.3,33.3) |  | 14187 (11971,16921) | 27.5 (23.2,32.8) |  | -1.4 (-5.1,2.1) |
| Central Asia | 25297 (21390,30480) | 46.2 (39,55.9) |  | 44278 (37279,53732) | 45.6 (38.6,55) |  | -1.3 (-4.5,2.2) |
| Central Europe | 91961 (79263,107720) | 65.8 (56.7,77) |  | 102432 (88116,120328) | 67.1 (57.4,78.8) |  | 2 (-0.7,4.6) |
| Central Latin America | 6623 (5510,8075) | 5.6 (4.7,6.7) |  | 14704 (12236,17650) | 5.6 (4.6,6.7) |  | -0.2 (-3.8,3.3) |
| Central Sub-Saharan Africa | 4420 (3728,5289) | 13.2 (11.2,15.9) |  | 11350 (9614,13814) | 12.5 (10.6,15) |  | -5.2 (-11.2,1.5) |
| East Asia | 64807 (54671,78247) | 5.6 (4.8,6.7) |  | 172201 (145043,206864) | 9.1 (7.7,10.9) |  | 61.2 (55.7,67.3) |
| Eastern Europe | 97793 (82909,118518) | 37 (31.5,44.8) |  | 101044 (85760,121703) | 35.4 (30,42.6) |  | -4.3 (-6.8,-1.8) |
| Eastern Sub to Saharan Africa | 12511 (10514,15078) | 11.3 (9.5,13.6) |  | 34879 (29436,42004) | 12.9 (10.9,15.4) |  | 13.9 (11.5,16.5) |
| High-income Asia Pacific | 59822 (51060,71293) | 30 (25.6,35.9) |  | 103656 (88507,124314) | 37.3 (31.6,45) |  | 24.4 (20.2,28.7) |
| High to income North America | 648147 (574642,742483) | 203.4 (180.3,233.5) |  | 997968 (872986,1147776) | 198.4 (172.5,231.7) |  | -2.4 (-5.6,0.4) |
| North Africa and Middle East | 70499 (59012,84300) | 29.2 (24.6,34.8) |  | 215400 (179465,259039) | 35.7 (29.9,42.9) |  | 22.3 (18.9,25.8) |
| Oceania | 279 (231,343) | 5.8 (4.8,7.2) |  | 642 (534,789) | 5.6 (4.6,6.8) |  | -4 (-9.5,2.2) |
| South Asia | 301261 (253266,365312) | 38.9 (33,46.9) |  | 802536 (676700,975731) | 46.2 (39.2,55.7) |  | 18.8 (16.9,20.7) |
| Southeast Asia | 20947 (17481,25167) | 5.6 (4.7,6.6) |  | 42506 (35567,51597) | 5.7 (4.8,6.9) |  | 2 (-0.7,4.6) |
| Southern Latin America | 23179 (19569,28040) | 48.9 (41.3,59.4) |  | 41020 (34731,50197) | 52.4 (44.3,64.4) |  | 7 (1.4,13.1) |
| Southern Sub to Saharan Africa | 5106 (4342,6116) | 14.4 (12.3,17.2) |  | 12192 (10352,14773) | 17.3 (14.8,20.7) |  | 19.7 (16.8,22.8) |
| Tropical Latin America | 15847 (13437,19020) | 12.8 (11,15.2) |  | 52828 (44696,63509) | 20.4 (17.3,24.4) |  | 59.2 (52.7,66) |
| Western Europe | 651533 (571491,737273) | 143 (125.8,162.6) |  | 929733 (806000,1081441) | 156.5 (134.5,181.9) |  | 9.5 (4.6,14.2) |
| Western Sub-Saharan Africa | 15303 (12871,18502) | 12.8 (10.8,15.5) |  | 46950 (39669,56938) | 15.8 (13.5,19.2) |  | 23.9 (21.3,26.8) |

Abbreviations: IBD, inflammatory bowel disease; ASPR, age standardized rate of prevalence.

**TableS5.The Disability to Adjusted Life Years (DALYs) ,age to standardized DALYs rate of IBD and their Percentage change from 1990 to 2021 at the global and regional levels.**

| **Location** | **1990** | |  | **1990 to 2021** | |  | **1990 to 2021** |
| --- | --- | --- | --- | --- | --- | --- | --- |
|  | **no(95% UI)** | **ASRs of DALYs per 100000(95% UI)** |  | **2021 No(95% UI)** | **ASRs of DALYs per 100000(95% UI)** |  | **Percentage change in the DALYs per 100000** |
| Global | 948861 (808101,1096717) | 21.5 (18.5,24.8) |  | 1510784 (1308508,1750363) | 18.1 (15.7,20.9) |  | -16.1 (-22,-9.8) |
| Andean Latin America | 3710 (2621,5137) | 10.6 (8,13.7) |  | 4056 (3243,5112) | 6.3 (5.1,8) |  | -40.2 (-56.3,-15.8) |
| Australasia | 7706 (5514,10323) | 34.5 (24.5,46.1) |  | 18181 (14279,22717) | 41.7 (31.9,53.8) |  | 21.1 (12.2,32.5) |
| Caribbean | 6475 (5487,7606) | 21.8 (18.7,25.3) |  | 7921 (6413,9847) | 15.5 (12.5,19.3) |  | -29.1 (-39.1,-18.4) |
| Central Asia | 13066 (10958,15115) | 21.4 (18.4,24.7) |  | 17039 (14249,20607) | 17.9 (15,21.6) |  | -16.2 (-26.5,-3.2) |
| Central Europe | 30676 (25949,36467) | 22.2 (18.8,26.4) |  | 33737 (28347,40508) | 20 (16.4,24.2) |  | -9.8 (-15.4,-4.1) |
| Central Latin America | 11772 (11222,12377) | 10.4 (10,10.8) |  | 26766 (24122,29560) | 10.4 (9.4,11.5) |  | 0.4 (-8.4,9.8) |
| Central Sub-Saharan Africa | 4269 (3000,5694) | 13 (9.3,17.1) |  | 10677 (7389,14923) | 12.6 (9,17.3) |  | -3.3 (-27.8,25.1) |
| East Asia | 167496 (120085,212704) | 18.3 (12.8,23) |  | 143569 (116156,180091) | 7.8 (6.3,9.6) |  | -57.7 (-68.7,-39.2) |
| Eastern Europe | 67526 (59874,76774) | 25.5 (22.6,29) |  | 60784 (54388,68505) | 20.1 (17.8,22.7) |  | -21.3 (-30.1,-13.4) |
| Eastern Sub to Saharan Africa | 14147 (10733,17906) | 13.2 (9.7,16.6) |  | 33535 (23245,43657) | 13.1 (9.1,17.3) |  | -0.7 (-21.1,21.9) |
| High-income Asia Pacific | 22939 (18874,27271) | 12.1 (10,14.3) |  | 26078 (20507,32420) | 8.4 (6.4,10.7) |  | -30.5 (-43.5,-15) |
| High to income North America | 143107 (113272,179200) | 44.1 (34.6,55.5) |  | 266271 (220857,319149) | 49.6 (40.2,60.6) |  | 12.4 (8.1,18.1) |
| North Africa and Middle East | 29875 (22461,40990) | 12.5 (9.9,16.6) |  | 65235 (52158,81440) | 11.8 (9.5,14.6) |  | -5.8 (-21.9,14) |
| Oceania | 551 (302,780) | 13.2 (7.2,18.9) |  | 884 (631,1235) | 8.5 (6.1,11.6) |  | -36 (-55.5,7.9) |
| South Asia | 146279 (114030,186335) | 19.5 (15,24.3) |  | 269363 (211770,341921) | 16.1 (12.7,20.4) |  | -17.3 (-33.1,-1) |
| Southeast Asia | 28473 (17974,36114) | 8.8 (5.3,11.8) |  | 40665 (30950,47973) | 6.1 (4.6,7.2) |  | -30.4 (-45.7,-7) |
| Southern Latin America | 7986 (6780,9515) | 17 (14.4,20.2) |  | 10421 (8332,13030) | 13 (10.3,16.4) |  | -23.2 (-31,-15.4) |
| Southern Sub to Saharan Africa | 4974 (3906,5968) | 13.1 (10,16.6) |  | 8962 (7397,10630) | 13.1 (10.9,15.5) |  | 0.3 (-18.3,22.5) |
| Tropical Latin America | 19416 (18414,20787) | 16.7 (15.9,17.9) |  | 43913 (40418,47689) | 17.2 (15.9,18.6) |  | 2.8 (-2.4,8.6) |
| Western Europe | 180624 (149913,216859) | 37.6 (30.7,45.7) |  | 318237 (270223,372594) | 42.9 (34.9,52.3) |  | 14.1 (9.8,18.7) |
| Western Sub-Saharan Africa | 37796 (25897,47096) | 28.8 (20,36.9) |  | 104491 (64783,142649) | 30.2 (19.8,40.7) |  | 4.9 (-13.8,29.5) |

Abbreviations: IBD, inflammatory bowel disease; DALYs, Disability-Adjusted Life Years; ASR, age standardized rate.

**TableS6.Global and Regional Level IBD Disability-Adjusted Life Years (DALYs), Age-Standardized Rates, and Age-Standardized APCs, 1990-2021**

|  | **Both** | |  | **Female** | |  | **Male** | |
| --- | --- | --- | --- | --- | --- | --- | --- | --- |
|  | **Segment** | **APC 95%CIs** |  | **Segment Start** | **APC 95%CIs** |  | **Segment** | **APC 95%CIs** |
| Incidence | 1990 to 1995 | 0.1569,(-0.0538,0.368) |  | 1990 to 1996 | 0.2574,(0.0856,0.4294) |  | 1990 to 1995 | 0.085,(-0.1101,0.2805) |
|  | 1995 to 2000 | 0.8893,(0.5916,1.1878) |  | 1996 to 1999 | 1.0552,(0.0406,2.08) |  | 1995 to 2000 | 1.0211,(0.7436,1.2993) |
|  | 2000 to 2005 | -0.2082,(-0.4996,0.084) |  | 1999 to 2005 | -0.1221,(-0.3448,0.1012) |  | 2000 to 2005 | -0.1976,(-0.4681,0.0736) |
|  | 2005 to 2010 | 1.5816,(1.2889,1.8752) |  | 2005 to 2010 | 1.7755,(1.4567,2.0952) |  | 2005 to 2010 | 1.3619,(1.0908,1.6336) |
|  | 2010 to 2021 | -0.6447,(-0.7103,-0.5791) |  | 2010 to 2021 | -0.679,(-0.7497,-0.6082) |  | 2010 to 2021 | -0.6074,(-0.6687,-0.5462) |
| Prevalence | 1990 to 1999 | 0.1413,(-0.1042,0.1784) |  | 1990 to 1997 | 0.1665,(0.0959,0.2371) |  | 1990 to 2000 | 0.1721,(0.1368,0.2074) |
|  | 1999 to 2005 | -0.2048,(-0.2941,-0.1153) |  | 1997 to 2005 | -0.2632,(-0.3326,-0.1938) |  | 2000 to 2005 | -0.0794,(-0.221,0.0623) |
|  | 2005 to 2010 | 1.5319,(1.4074,1.6564) |  | 2005 to 2010 | 1.6962,(1.5356,1.857) |  | 2005 to 2010 | 1.3307,(1.1896,1.472) |
|  | 2010 to 2014 | -1.8022,(-1.9983,-1.6058) |  | 2010 to 2014 | -1.8521,(-2.1065,-1.597) |  | 2010 to 2014 | -1.7397,(-1.9659,-1.5129) |
|  | 2014 to 2019 | -1.3104,(-1.4463,-1.1743) |  | 2014 to 2019 | -1.3722,(-1.5475,-1.1965) |  | 2014 to 2019 | -1.2385,(-1.3926,-1.0842) |
|  | 2019 to 2021 | -0.3354,(-0.7795,0.1106) |  | 2019 to 2021 | -0.3596,(-0.9263,0.2102) |  | 2019 to 2021 | -0.2956,(-0.7999,0.2113) |
| Deaths | 1990 to 1998 | -0.2827,(-0.6298,0.0656) |  | 1990 to 1998 | -0.3574,(-0.7463,0.0329) |  | 1990 to 1994 | 0.639,(0.1379,1.1426) |
|  | 1998 to 2003 | 1.5429,(0.5876,2.5072) |  | 1998 to 2003 | 1.9817,(0.9541,3.0199) |  | 1994 to 1997 | -1.375,(-2.9718,0.2481) |
|  | 2003 to 2012 | -0.5175,(-0.8091,-0.225) |  | 2003 to 2012 | -0.6005,(-0.9115,-0.2885) |  | 1997 to 2004 | 1.0059,(0.7537,1.2586) |
|  | 2012 to 2021 | -1.7513,(-1.9979,-1.504) |  | 2012 to 2021 | -1.9667,(-2.2477,-1.6849) |  | 2004 to 2009 | -0.9222,(-1.317,-0.5258) |
|  |  | | | | |  | 2009 to 2012 | 0.4213,(-0.6423,1.4962) |
|  |  |  |  |  |  |  | 2012 to 2021 | -1.5475,(-1.6602,-1.4346) |
| DALYs | 1990 to 1994 | 0.2358,(0.0014,0.4707) |  | 1990 to 1994 | -0.1565,(-0.4717,0.1597) |  | 1990 to 1994 | 0.5966,(0.2747,0.9197) |
|  | 1994 to 1997 | -1.3108,(-2.0325,-0.5837) |  | 1994 to 1997 | -1.2422,(-2.1871,-0.2881) |  | 1994 to 1997 | -1.4132,(-2.4237,-0.3922) |
|  | 1997 to 2002 | 0.1607,(-0.0704,0.3923) |  | 1997 to 2002 | 0.3183,(0.0392,0.5981) |  | 1997 to 2001 | 0.0214,(-0.4885,0.534) |
|  | 2002 to 2012 | -0.2863,(-0.3526,-0.2199) |  | 2002 to 2012 | -0.2052,(-0.2786,-0.1318) |  | 2001 to 2011 | -0.3143,(-0.4078,-0.2207) |
|  | 2012 to 2015 | -1.8964,(-2.6119,-1.1757) |  | 2012 to 2015 | -2.0497,(-2.8114,-1.282) |  | 2011 to 2021 | -1.2503,(-1.3304,-1.1702) |
|  | 2015 to 2021 | -1.1451,(-1.2688,-1.0212) |  | 2015 to 2021 | -1.1582,(-1.291,-1.0252) |  |  |  |
| YLDs | 1990 to 1999 | 0.1162,(0.0801,0.1525) |  | 1990 to 1997 | 0.109,(0.0141,0.204) |  | 1990 to 2000 | 0.1704,(0.1345,0.2063) |
|  | 1999 to 2005 | -0.1916,(-0.2802,-0.103) |  | 1997 to 2005 | -0.2403,(-0.3355,-0.1451) |  | 2000 to 2005 | -0.0877,(-0.2328,0.0575) |
|  | 2005 to 2010 | 1.552,(1.4247,1.6795) |  | 2005 to 2010 | 1.6646,(1.436,1.8936) |  | 2005 to 2010 | 1.349,(1.2025,1.4958) |
|  | 2010 to 2014 | -1.7643,(-1.9594,-1.5687) |  | 2010 to 2018 | -1.5982,(-1.6924,-1.5039) |  | 2010 to 2014 | -1.7544,(-1.9794,-1.5288) |
|  | 2014 to 2019 | -1.3151,(-1.4407,-1.1894) |  | 2018 to 2021 | -0.5954,(-0.9476,-0.2419) |  | 2014 to 2019 | -1.263,(-1.4073,-1.1185) |
|  | 2019 to 2021 | -0.3987,(-0.7953,-0.0005) |  |  |  |  | 2019 to 2021 | -0.3493,(-0.8074,0.111) |
| YLLs | 1990 to 1994 | 0.2591,(-0.1992,0.7196) |  | 1990 to 1998 | -0.996,(-1.3414,-0.6493) |  | 1990 to 1994 | 0.811,(0.3976,1.2261) |
|  | 1994 to 1997 | -1.9838,(-3.3445,-0.6041) |  | 1998 to 2003 | 0.6178,(-0.2301,1.4729) |  | 1994 to 1997 | -2.1255,(-3.4288,-0.8047) |
|  | 1997 to 2004 | 0.1848,(-0.04,0.4102) |  | 2003 to 2012 | -0.894,(-1.1302,-0.6571) |  | 1997 to 2004 | -0.108,(-0.3126,0.097) |
|  | 2004 to 2009 | -1.2644,(-1.6225,-0.9051) |  | 2012 to 2021 | -1.3977,(-1.5906,-1.2044) |  | 2004 to 2009 | -1.2474,(-1.5766,-0.9171) |
|  | 2009 to 2012 | -0.3676,(-1.4059,0.6816) |  |  | |  | 2009 to 2012 | -0.2045,(-1.0822,0.681) |
|  | 2012 to 2021 | -1.4088,(-1.511,-1.3064) |  |  |  |  | 2012 to 2021 | -1.3067,(-1.4014,-1.2119) |

Abbreviations: IBD, inflammatory bowel disease; ASRs, age-standardized rates; ASMR, age-standardized mortality rate; ASIR, age-standardized incidence rate; ASPR, age-standardized prevalence rate; DALYs, disability-adjusted life-years; YLDs, years lived with disability; YLLs, years of life lost; APC: Annual Percent Change; CI: Confidence Interval

**TableS7.Global and SDI-stratified age-standardized rates (ASRs) of incidence for inflammatory bowel disease in 2020, 2030, 2040, and 2050, with projected values and corresponding 95% uncertainty intervals.**

|  | **Location** | **Estimate in 2020**  **(95% CIs)** | **Estimate in 2030**  **(95% CIs)** | **Estimate in 2040**  **(95% CIs)** | **Estimate in 2050**  **(95% CIs)** |
| --- | --- | --- | --- | --- | --- |
| **ASMR** | Global | 0.54 (0.53,0.55) | 0.48 (0.36,0.60) | 0.42 (0.14,0.70) | 0.36 (-0.08,0.79) |
|  | High SDI | 0.90 (0.88,0.92) | 0.76 (0.19,1.33) | 0.66 (-0.76,2.07) | 0.54 (-1.60,2.68) |
|  | High-middle SDI | 0.71 (0.69,0.74) | 0.65 (0.42,0.88) | 0.57 (0.01,1.13) | 0.47 (-0.38,1.33) |
|  | Middle SDI | 0.26 (0.26,0.27) | 0.25 (0.17,0.33) | 0.24 (0.03,0.46) | 0.24 (-0.14,0.62) |
|  | Low-middle SDI | 0.32 (0.31,0.33) | 0.28 (0.22,0.33) | 0.24 (0.11,0.37) | 0.22 (0.01,0.42) |
|  | Low SDI | 0.56 (0.54,0.58) | 0.50 (0.39,0.62) | 0.45 (0.18,0.72) | 0.41 (-0.02,0.84) |
| **ASIR** | Global | 4.46 (4.44,4.48) | 4.35 (3.39,5.31) | 4.19 (1.59,6.78) | 4.04 (-0.56,8.63) |
|  | High SDI | 13.40 (13.30,13.50) | 13.40 (9.58,17.22) | 12.77 (2.62,22.93) | 11.88 (-5.42,29.18) |
|  | High-middle SDI | 6.64 (6.56,6.72) | 6.46 (4.83,8.09) | 6.06 (1.90,10.23) | 5.64 (-1.41,12.69) |
|  | Middle SDI | 1.93 (1.91,1.95) | 2.09 (1.47,2.72) | 2.19 (0.33,4.05) | 2.21 (-1.25,5.67) |
|  | Low-middle SDI | 3.51 (3.47,3.54) | 3.51 (2.76,4.27) | 3.50 (1.37,5.62) | 3.46 (-0.40,7.32) |
|  | Low SDI | 3.38 (3.33,3.43) | 3.41 (2.75,4.07) | 3.42 (1.60,5.24) | 3.40 (0.11,6.69) |
| **ASPR** | Global | 45.21 (45.15,45.28) | 42.47 (32.03,52.91) | 39.89 (12.47,67.31) | 37.83 (-9.80,85.46) |
|  | High SDI | 152.95 (152.62,153.29) | 152.16 (94.92,209.41) | 146.33 (-8.05,300.72) | 138.17 (-129.04,405.37) |
|  | High-middle SDI | 64.00 (63.74,64.26) | 63.36 (43.50,83.21) | 60.32 (9.50,111.14) | 56.88 (-29.86,143.62) |
|  | Middle SDI | 15.85 (15.78,15.91) | 16.62 (12.51,20.74) | 16.94 (5.04,28.85) | 16.67 (-4.89,38.24) |
|  | Low-middle SDI | 26.79 (26.69,26.89) | 26.15 (21.19,31.12) | 25.27 (11.60,38.95) | 24.28 (0.07,48.50) |
|  | Low SDI | 26.05 (25.89,26.21) | 26.19 (21.56,30.82) | 26.31 (13.19,39.43) | 26.31 (2.22,50.40) |
| **DALYs** | Global | 18.31 (18.26,18.35) | 17.09 (13.85,20.32) | 15.15 (7.57,22.73) | 12.82 (1.27,24.37) |
|  | High SDI | 40.18 (40.01,40.35) | 35.55 (22.42,48.67) | 30.68 (-1.63,62.99) | 25.31 (-23.80,74.43) |
|  | High-middle SDI | 25.54 (25.38,25.71) | 23.50 (16.54,30.46) | 20.26 (3.98,36.55) | 16.42 (-7.51,40.36) |
|  | Middle SDI | 8.82 (8.78,8.87) | 8.94 (6.80,11.08) | 8.74 (3.13,14.34) | 8.16 (-1.30,17.61) |
|  | Low-middle SDI | 12.78 (12.72,12.85) | 11.72 (9.72,13.72) | 10.55 (5.56,15.53) | 9.30 (1.28,17.32) |
|  | Low SDI | 20.81 (20.66,20.96) | 18.92 (15.30,22.55) | 17.22 (8.23,26.22) | 15.65 (0.81,30.50) |
| **YLDs** | Global | 6.85 (6.82,6.87) | 6.45 (4.89,8.02) | 6.05 (1.92,10.18) | 5.69 (-1.44,12.82) |
|  | High SDI | 22.41 (22.29,22.54) | 22.05 (14.07,30.04) | 20.78 (-0.48,42.04) | 19.01 (-16.73,54.74) |
|  | High-middle SDI | 9.77 (9.66,9.87) | 9.56 (6.61,12.52) | 8.97 (1.49,16.44) | 8.27 (-4.23,20.78) |
|  | Middle SDI | 2.53 (2.51,2.56) | 2.66 (1.94,3.37) | 2.71 (0.63,4.78) | 2.65 (-1.10,6.40) |
|  | Low-middle SDI | 4.34 (4.31,4.38) | 4.17 (3.34,4.99) | 3.96 (1.74,6.19) | 3.73 (-0.12,7.59) |
|  | Low SDI | 4.32 (4.27,4.38 | 4.27 (3.45,5.08) | 4.21 (1.99,6.44) | 4.14 (0.15,8.12) |
| **YLLs** | Global | 11.46 (11.43,11.49) | 10.58 (8.31,12.86) | 9.36 (4.00,14.73) | 8.07 (-0.26,16.41) |
|  | High SDI | 17.77 (17.66,17.88) | 15.05 (7.00,23.11) | 12.34 (-6.61,31.29) | 9.65 (-17.71,37.01) |
|  | High-middle SDI | 15.79 (15.66,15.92) | 13.50 (7.69,19.31) | 11.12 (-2.20,24.43) | 8.74 (-10.40,27.87) |
|  | Middle SDI | 6.29 (6.25,6.33) | 6.15 (4.23,8.06) | 5.93 (0.84,11.02) | 5.63 (-3.17,14.44) |
|  | Low-middle SDI | 8.43 (8.37,8.48) | 7.47 (6.14,8.80) | 6.58 (3.38,9.78) | 5.76 (0.67,10.85) |
|  | Low SDI | 16.46 (16.32,16.59) | 14.52 (11.53,17.51) | 12.96 (5.74,20.17) | 11.61 (-0.10,23.33) |

Abbreviations: ASRs, age-standardized rates; ASMR, age-standardized mortality rate; ASIR, age-standardized incidence rate; ASPR, age-standardized prevalence rate; DALYs, disability-adjusted life-years; YLDs, years lived with disability; YLLs, years of life lost; SDI, Socio-demographic Index; CI: Confidence Interval
